# Comparison and Phylogenetic Analysis of Chloroplast Genomes of Three Medicinal and Edible *Amomum* Species

**DOI:** 10.3390/ijms20164040

**Published:** 2019-08-19

**Authors:** Yingxian Cui, Xinlian Chen, Liping Nie, Wei Sun, Haoyu Hu, Yulin Lin, Haitao Li, Xilong Zheng, Jingyuan Song, Hui Yao

**Affiliations:** 1 Key Lab of Chinese Medicine Resources Conservation, State Administration of Traditional Chinese Medicine of the People’s Republic of China, Institute of Medicinal Plant Development, Chinese Academy of Medical Sciences & Peking Union Medical College, Beijing 100193, China; 2 Engineering Research Center of Chinese Medicine Resources, Ministry of Education, Beijing 100193, China; 3 Institute of Chinese Materia Medica, China Academy of Chinese Medical Sciences, Beijing 100700, China; 4 Yunnan Branch, Institute of Medicinal Plant Development, Chinese Academy of Medical Sciences & Peking Union Medical College, Jinghong 666100, China; 5 Hainan Branch, Institute of Medicinal Plant Development, Chinese Academy of Medical Sciences & Peking Union Medical College, Wanning 571533, China

**Keywords:** *Amomum villosum*, *A. villosum* var. *xanthioides*, *A. longiligulare*, chloroplast genome, comparative analysis, species authentication, phylogenetic analysis

## Abstract

*Amomum villosum* is an important medicinal and edible plant with several pharmacologically active volatile oils. However, identifying *A. villosum* from *A. villosum* var. *xanthioides* and *A. longiligulare* which exhibit similar morphological characteristics to *A. villosum*, is difficult. The main goal of this study, therefore, is to mine genetic resources and improve molecular methods that could be used to distinguish these species. A total of eight complete chloroplasts (cp) genomes of these *Amomum* species which were collected from the main producing areas in China were determined to be 163,608–164,069 bp in size. All genomes displayed a typical quadripartite structure with a pair of inverted repeat (IR) regions (29,820–29,959 bp) that separated a large single copy (LSC) region (88,680–88,857 bp) from a small single copy (SSC) region (15,288–15,369 bp). Each genome encodes 113 different genes with 79 protein-coding genes, 30 tRNA genes, and four rRNA genes. More than 150 SSRs were identified in the entire cp genomes of these three species. The Sanger sequencing results based on 32 *Amomum* samples indicated that five highly divergent regions screened from cp genomes could not be used to distinguish *Amomum* species. Phylogenetic analysis showed that the cp genomes could not only accurately identify *Amomum* species, but also provide a solid foundation for the establishment of phylogenetic relationships of *Amomum* species. The availability of cp genome resources and the comparative analysis is beneficial for species authentication and phylogenetic analysis in *Amomum*.

## 1. Introduction

**Figure 1:**
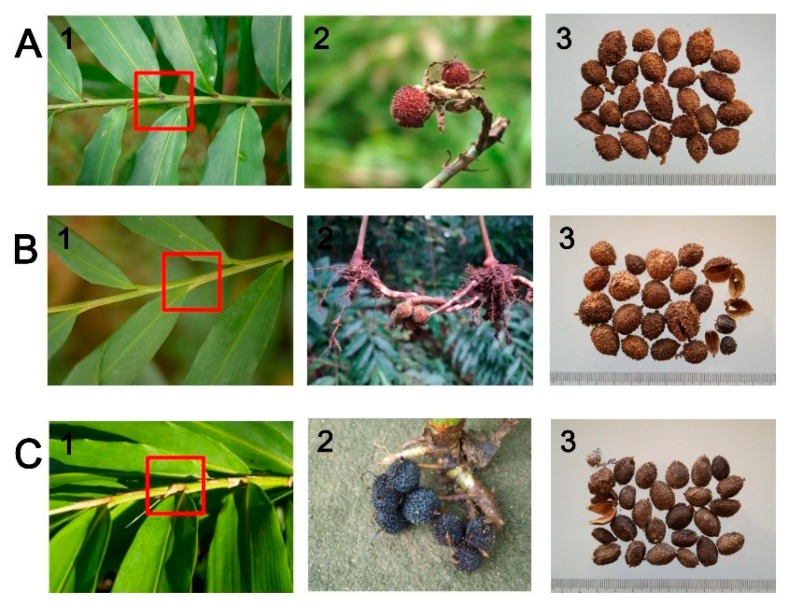
Commercial dried samples and plant materials of *A. villosum* (**A**), *A. villosum* var. *xanthioides* (**B**) and *A. longiligulare* (**C**). In picture 1, differences in the size and color of ligules are highlighted in red boxes. In picture 2, fresh fruits were removed from soil and photographed. In picture 3, amomi fructus (dried fruits) were collected from herb markets and photographed.

*Amomum villosum* Lour., which belongs to the monophyletic Zingiberaceae family, is a valuable medicinal plant in China with a history of more than 1300 years [1]. As a plant with both medicinal and edible functions, its ripe fruits, called amomi fructus, are not only widely used clinically but also often used as a type of condiment. In China, amomi fructus is one of the “Four Major Southern Medicines” and plays important roles in clinical treatment, such as warming the spleen, eliminating dampness and diarrhea, promoting appetite, and preventing miscarriage [2]. Amomi fructus possesses potential therapeutic effect for inflammatory bowel disease [3], radical scavenging [4], and has analgesic and anti-inflammatory effects [5], which have recently gained increasing attention. Three authentic plant sources of amomi fructus have been recorded in the Chinese pharmacopoeia, namely, *A. villosum* Lour., *A. villosum* Lour. var. *xanthioides* T. L. Wu et Senjen and *A. longiligulare* T. L. Wu [2]. However, research has shown that the content of the active ingredient (borneol acetate) is significantly higher in *A. villosum* than in *A. villosum* var. *xanthioides* and *A. longiligulare*, which is consistent with the statement that the effects of *A. villosum* are better than those of the other two species [6,7], resulting in the high value of *A. villosum* in clinical use. Given the special flower structure of *A. villosum*, the plant exhibits difficulty in self-pollination and must rely on a few kinds of insects or artificial pollination. This pollination characteristic greatly reduces the yield of *A. villosum* [6]. Given their high economic and medicinal values, the ripe fruits of *A. villosum* var. *xanthioides* and *A. longiligulare*, which exhibit high similarity in morphological characters and confusing Chinese names with *A. villosum*, are often used as adulterants or contaminants of *A. villosum* in the market (Figure 1) [8,9]. In addition, the appearances of these three *Amomum* plants are extremely similar, and there are only a few differences on their leaves, which have been marked in Figure 1 with red boxes. Generally speaking, the ligules of *A. villosum* are purple and relatively short; the ligules sizes of *A. villosum* var. *xanthioides* are almost similar to those of *A. villosum*, but the color is green; and those of *A. longiligulare* are also purple in color, but their size is longer than the other two. The mixture of *Amomum* species severely impacts efficient market processing and drug safety. ITS [10], ITS2 [9], randomly amplified polymorphic DNA [11], and single nucleotide polymorphisms (SNPs) [12] have been used to identify these three species, and some progress has been made. In recent years, the chloroplast (cp) genomes have shown great potential for species authentication, especially between closely related species [13,14], breeding, and phylogenetic analysis. Thus, developing cp genomic resources of these three *Amomum* species is not only beneficial in accurately identifying the closely related species, but it can also greatly contribute to the improvement of cp genetic engineering. In addition, up to now, only two *Amomum* cp genome sequences—*A. kravanh* and *A. compactum*—have been reported. So, the availability of complete cp genome sequences of these three *Amomum* species will be helpful in revealing the evolutionary relationships and phylogenetic position of species in commelinids.

Cp is an organelle specialized for providing essential energy for growth and reproduction of plants, which converts solar energy into chemical energy and releases oxygen [15]. A typical angiosperm cp genome consists of two inverted repeats (IRs) which are separated by a large single copy (LSC) and a small single copy (SSC), and encodes key proteins for photosynthesis and other necessary metabolic processes for plants in response to environmental stresses, such as drought, salt, heat, and light [16]. Cp genomes generally encode 120–130 genes with lengths of 120–170 kb [17]. Compared with the nuclear genome, the cp genome is smaller and employs multiple copies that can improve the expression level of the target gene [18]. Cp genome sequences are known for their highly conserved gene order and content. However, large-scale genome rearrangements, gene transfers, and gene insertions and losses have been identified in previous studies [19,20,21]. Over the past years, cp genomes have been shown to be an efficient tool to reveal phylogenetic relationships [20], identify the related species as a super-barcode [13,14], and develop cp genetic engineering [22]. With the development of sequencing technologies, the number of cp genome sequences recorded in the National Center for Biotechnology Information (NCBI) has increased dramatically. However, for the whole plant community, the total number of plants that have determined cp genome sequences is still insufficient. As far as medicinal plants are concerned, according to the national survey on Chinese material medical resources, there are more than 11,146 known medicinal plants in China alone. However, up to now, approximately 3300 cp genome sequences of plants have been recorded in NCBI, and this proportion is still small. In order to further develop cp genetic resources, more plant cp genome data need to be developed.

In this study, eight complete cp genomes of *A. villosum*, *A. villosum* var. *xanthioides* and *A. longiligular* were determined. Differences in their essential characteristics and repeat sequences were revealed. Intraspecific and interspecific comparative analyses among the *Amomum* genus were conducted to discover highly divergent regions for species authentication. Thirty-two samples of eight *Amomum* species were collected to assess the species discriminatory power for these highly divergent regions. Furthermore, a phylogenetic tree was constructed to identify *Amomum* species and reveal their phylogenetic positions.

## 2. Results and Discussion

### 2.1. Genome Length and Features

**Figure 2:**
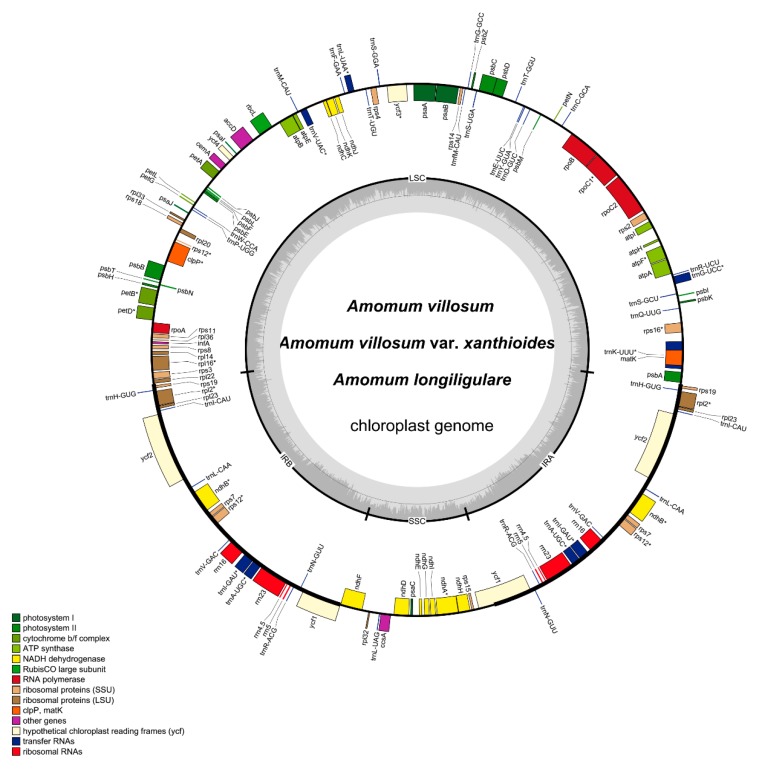
Gene map of the complete cp genomes of *A. villosum*, *A. villosum* var. *xanthioides* and *A. longiligulare*. Only one map is shown here because the differences among the three species are negligible, and the gene maps of the three cp genomes are almost identical. Genes of different functional groups are separated by color. Genes inside the circle are transcribed clockwise, whereas those on the outside are transcribed counter-clockwise. The dark grey area in the inner circle corresponds to GC content, whereas the light grey area corresponds to AT content.

A total of eight complete cp genomes of *Amomum* species were determined and submitted to GenBank (accession number: MH161416-18 and MN067431-35), which comprised four (Sample numbers: Y17085, Y17089, Y19017, and Y19021), two (Sample numbers: Y17088 and Y19018), and two (Sample numbers: Y19019 and Y19020) individual plants of *A. villosum*, *A. villosum* var. *xanthioides* and *A. longiligulare*, respectively. All three species studied here included individuals from the main producing areas in China [23]. In total, 34,775,994–49,499,932 raw reads were generated, and 257,464–780,870 reads were finally assembled to generate complete cp genomes with 232–714× sequencing depth. The complete cp genomes of the eight species from *A. villosum*, *A. villosum* var. *xanthioides* and *A. longiligulare* were determined to be 163,608-164,069 bp in size (Table 1). All displayed a typical quadripartite structure with a pair of IR regions (29,820–29,959 bp) that separated an LSC region (88,680–88,857 bp) from an SSC region (15,288–15,369 bp) (Table 1 and Figure 2). The overall GC content of the three species was 36.0–36.1% (Table 1). The GC content was unevenly distributed in the cp genome of these species. The GC content was the highest (41.0%) in the IR regions, the lowest (29.9–30.1%) in the SSC region, and approximately 33.7% in the LSC region (Table 1). These values were similar to most other reported cp genomes [19,24]. The high GC content of the IR region could be due to the four ribosomal RNA (rRNA) genes with a reduced number of duplicated AT nucleotides [25] and may be one of the important factors that cause the IR region to be more conservative than the LSC and SSC regions [26]. This phenomenon is also evident in many other angiosperms, such as *A. kravanh* [27], *A. compactum* [28], *Scutellaria baicalensis* [25] and *Schisandra chinensis* [29]. The AT representation at the third codon position (71.2~71.3%) was higher than that at the first (55.4%) and second (62.6%) positions in the protein-coding regions (CDS) of these species (Table 1). This bias was used as one principle to discriminate cp DNA from nuclear and mitochondrial DNA.

The complete cp genomes of these three species encode 113 different genes, including 79 protein-coding genes, 30 tRNA genes, and four rRNA genes (Table 2). A total of 20 genes were duplicated in the IR regions, resulting in a total of 133 genes located in the complete cp genomes (Table 2). The gene distribution in these three cp genomes was exactly the same: the LSC regions encoded 58 protein-coding genes and 23 tRNA genes, and the SSC regions contained 11 protein-coding genes and one tRNA gene. Moreover, eight protein-coding genes, eight tRNA genes and all four rRNA genes were duplicated in the IR regions.

In eukaryotic and semi-prokaryotic systems, gene expression occurs in nuclear-cytosolic and organelle compartments, respectively. Introns contribute greatly to the regulation of gene expression, and previous studies proven that intron can improve expression levels of exogenous genes in eukaryotic genomes [30]. Introns can accumulate more mutations than exons, and they maybe contain “old code”, which is the part of a gene that has lost its function during evolution [31,32,33]. A total of 18 intron-containing genes were found in the cp genomes of these three species, 15 of which contained one intron, and two (*ycf3* and *clpP*) contained two introns (Figure 1, Table 2 and Table S1). The *rps12* gene was a special trans-spliced gene with the 5′ end located in the LSC region and the duplicated 3′ ends in the IR regions. Among the 18 intron-containing genes, 12 were present in LSC regions, only one was present in the SSC region, and five were duplicated in the IR regions. The *matK* gene was contained in the intron of *trnK-UUU* gene, which showed the largest intron with more than 2500 bp.

### 2.2. Codon Usage

Codon usage patterns of coding sequences for *A. villosum*, *A. villosum* var. *xanthioides* and *A. longiligulare* were calculated based on the relative synonymous codon usage (RSCU) value [34]. We defined that the codon whose RSCU values were >1.00 was used more frequently, and vice versa. All protein-coding genes of *A. villosum* were encoded by 27,730 codons, *A. longiligulare* was encoded by 27,720 codons, and that of *A. villosum* var. *xanthioides* was 27,726–27,732 codons (Table S2). Like most angiosperms, leucine is the most prevalent amino acid in the cp genomes of *A. villosum* (2853, 10.3%), *A. villosum* var. *xanthioides* (2844–2851, 10.3%), and *A. longiligulare* (2855, 10.3%). Conversely, cysteine, with 316–317 (1.1%) codons, was the least frequent amino acid in the cp genomes of these three species. Except for *trnL-CAA* encoded by UUG, amino acid codon (RSCU > 1) in the cp genomes of three species preferentially showed A- or U-endings, which corresponded to the mentioned results that were calculated based on the cp genome sequences.

### 2.3. SSRs Analyses and Repeat Structures

**Figure 3:**
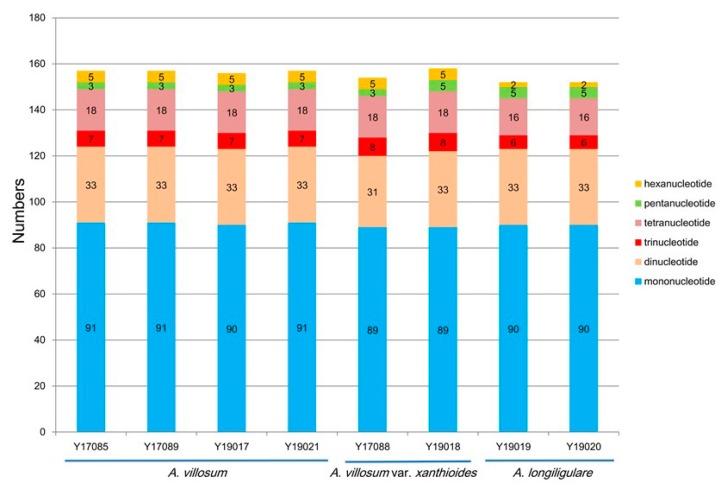
Analysis of simple sequence repeats (SSRs) in the cp genomes of three *Amomum* species.

Simple sequence repeats (SSRs), also called microsatellites, are short and tandem repeat DNA sequences with sizes of 1–6 bp [35] that are widely distributed throughout the cp genome and are usually used as important molecular markers for species authentication [36,37]. Here, we analyzed the distribution and the type of SSRs contained in the cp genomes of *A. villosum*, *A. villosum* var. *xanthioides* and *A. longiligulare*. A total of 157 SSRs were identified in the whole cp genome of *A. villosum*, including 91 mono-, 33 di-, 7 tri-, 18 tetra-, three penta-, and five hexa-nucleotide SSRs (Table S3 and Figure 3). Among these SSRs, 29 (18.47%) were contained in the CDS (Table S4). A total of 154 (Y17088) or 158 (Y19018) SSRs were present in the whole cp genomes of *A. villosum* var. *xanthioides* and included 89 mono-, 31 (Y17088) or 33 (Y19018) di-, eight tri-, 18 tetra-, three (Y17088) or five (Y19018) penta-, and five hexa-nucleotide SSRs (Table S3 and Figure 3). The total number of identified SSRs in the CDS was 29 (Y17088) or 31 (Y19018) (18.83%) (Table S4). For *A. longiligulare*, there were 152 SSRs that comprised 90 mono-, 33 di-, six tri-, 16 tetra-, five penta-, and two hexa-nucleotide SSRs (Table S3 and Figure 3). Our results, show in Table S3, were consistent with the finding [19,25] that SSRs appeared more frequently in the LSC regions than in the SSC and IR regions. The overwhelming majority of mononucleotide repeats were composed of A or T (Table S3). All SSR types are listed in Table S3. Among all SSR types, A and T were always the most frequently used bases, which was in line with previous findings [24,27] that cp SSRs are generally composed of short polyadenine or polythymine repeats, resulting from the bias toward A and T of cp genomes. Cp SSRs have been widely used as molecular markers for the population genetic structure and phylogeographic study of some species [38,39,40] due to high substitution rates. And cp SSRs markers can be applied as complementary tools of nuclear markers to determine species identification and genetic relationships among closely related species [41,42]. Therefore, the availability of cp SSRs is of great benefit to develop useful molecular markers for the studies of genetic diversity, population structure, evolutionary studies, molecular identification and other further investigations of *Amomum* species.

**Figure 4:**
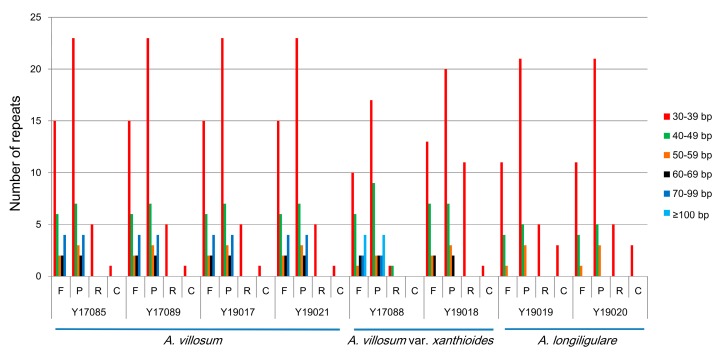
Repeat sequences in the three cp genomes. F, P, R, and C indicate the repeat types F (forward), P (palindrome), R (reverse), and C (complement), respectively.

Except for SSRs, some repeat structures with a length ≥30 bp are known as long repeat sequences, including forward repeats (F), palindromic repeats (P), reverse repeats (R), and complement repeats (C). These repeat structures promote the rearrangement of the cp genome and increase the population’s genetic diversity [29]. We analyzed the repeat structures of these three *Amomum* species. A total of 74 long repeats were present in the cp genome of *A. villosum*, as follows: 29 forward repeats, 39 palindromic repeats, five reverse repeats, and 1 complement repeat (Figure 4). A total of 63 (Y17088) or 68 (Y19018) long repeats were identified in the cp genome of *A. villosum* var. *xanthioides* including 25 (Y17088) or 24 (Y19018) forward repeats, 36 (Y17088) or 32 (Y19018) palindromic repeats, two (Y17088) or 11 (Y19018) reverse repeats and zero (Y17088) or one (Y19018) compliment repeats. As for the cp genomes of *A. longiligulare*, there were 53 long repeats, including 16 forward repeats, 29 palindromic repeats, five reverse repeats, and three complement repeats. In all three species, the majority of these repeats showed lengths between 30 and 39 bp. Only *A. villosum* var. *xanthioides* (Y17088) exhibited eight long repeats more than 100 bp in size (Figure 4).

### 2.4. Interspecific Comparison

From the above analysis, we can see that intraspecific differences between the cp genomes of four individuals of *A. villosum*, two individuals of *A. villosum* var. *xanthioides* and two individuals of *A. longiligulare* were very small. Here, in each of the three species, one of the cp genomes was used for interspecies comparative analyses within *Amomum* species *A. villosum* (Y17085), *A. villosum* var. *xanthioides* (Y17088), and *A. longiligulare* (Y19019).

**Figure 5:**
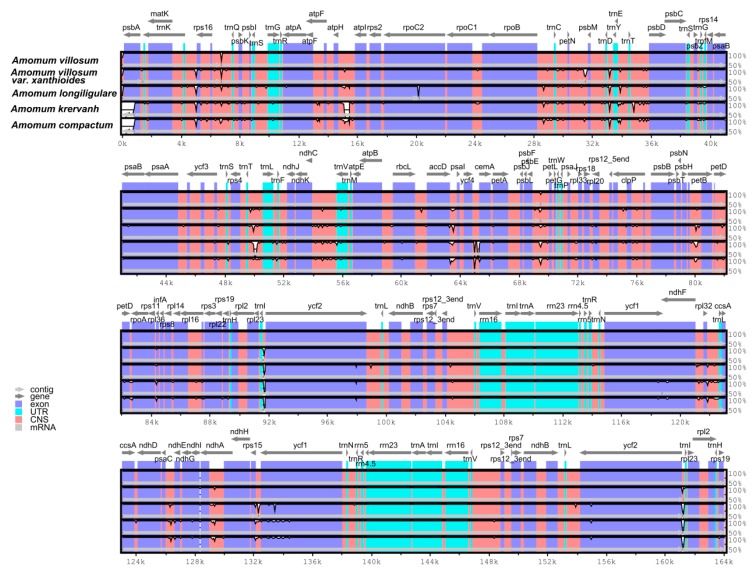
Sequence identity plot comparing the five cp genomes in *Amomum* species with *A. villosum* as a reference by using mVISTA. Grey arrows and thick black lines above the alignment indicate genes with their orientation and the position of the inverted repeats (IRs), respectively. A cut-off of 70% identity was used for the plots, and the Y-scale represents the percent identity ranging from 50% to 100%.

Interspecific comparisons between five *Amomum* species (*A. villosum*, *A. villosum* var. *xanthioides*, *A. longiligulare*, *A. krervanh* and *A. compactum*) were conducted using mVISTA software with the annotated cp genome of *A. villosum* as a reference (Figure 5). Among the five *Amomum* species, *A. villosum* exhibited the longest cp genome size and was closely followed by *A. villosum* var. *xanthioides* and *A. longiligulare*, and *A. krervanh* was the smallest one with the size of 162,766 bp [27]. The common features of most angiosperms in the IR regions were more conserved than in the LSC and SSC regions, and the four rRNA (*rrn4.5*, *rrn5*, *rrn16*, and *rrn23*) genes were the most conserved in the two IR regions. The non-coding regions were more divergent than the coding regions. The cp genomes of *A. villosum*, *A. villosum* var. *xanthioides* and *A. longiligulare* showed little difference from one another. Comparing *A. krervanh* and *A. compactum*, the highly divergent regions among these five cp genomes mainly occurred in the intergenic regions, including *atpH-atpI*, *trnD*-*trnY*, *accD-psaI*, *ycf4-cemA* and *trnI-ycf2*, which may be potential molecular markers for species authentication.

**Figure 6:**
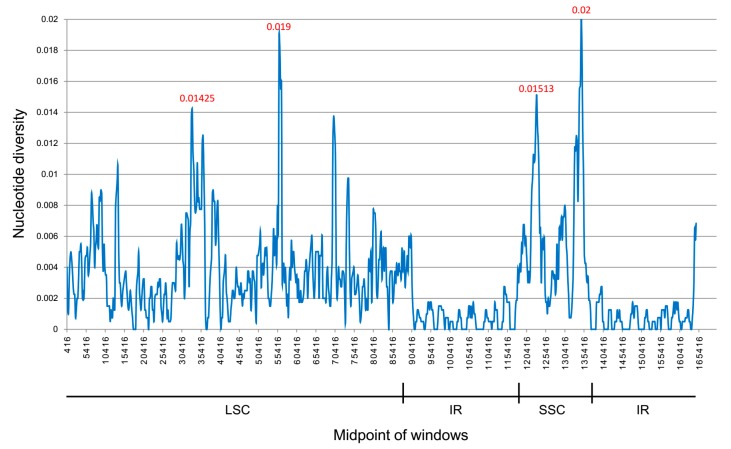
Sliding window analysis based on the cp genomes of five *Amomum* species. Window length: 800 bp; step size: 200 bp. *X*-axis: position of the midpoint of a window. *Y*-axis: nucleotide diversity of each window.

DNA polymorphism analyses were executed to detect highly variable regions and to show divergence at the sequence level in the cp genomes of the five *Amomum* species. The average value of nucleotide variability (PI) of all the five *Amomum* species was 0.00306. Results shown in Figure 6 also suggest that IR regions were less divergent than the LSC and SSC regions. A total of four mutational hotspots that showed remarkably high values of PI (≥0.014) were observed. As shown in Figure 6, mutational hotspots within these *Amomum* species were commonly located in the LSC and the SSC regions, which was in line with the result from mVISTA.

### 2.5. Species Authentication Analyses Based on cp Highly Divergent Regions

In order to assess the species discriminatory power of the highly divergent regions (*atpH-atpI*, *trnD*-*trnY*, *accD-psaI*, *ycf4-cemA*, and *trnI-ycf2*), a total of 32 samples from eight *Amomum* species (*A. villosum*, *A. villosum* var. *xanthioides*, *A. longiligulare*, *A. chinense*, *A. compactum*, *A. tsaoko*, *A. koenigii*, and *A. maximum*) were collected. Information about sample numbers and locations are listed in Table S5. To develop identification markers, specific primers were designed against conserved regions of *atpH-atpI*, *trnD-trnY*, *accD-psaI*, *ycf4-cemA*, and *trnI-ycf2* (Table S6). All highly divergent regions except *atpH*-*atpI* were amplified successfully (Figure S1). For *atpH*-*atpI*, we designed two pairs of specific primers, but amplification was still unsuccessful. Amplified fragments from all tested *Amomum* samples were sequenced, and high-quality sequences were used to construct an NJ phylogenetic tree (Figure S2) to exhibit the species discriminatory power of the four highly divergent regions. The results showed that *trnD*-*trnY* and *trnI-ycf2* could not distinguish these eight *Amomum* species. *accD-psaI* could differentiate *A. villosum*, *A. villosum* var. *xanthioides* and *A. longiligulare* from their closely related species- but could not distinguish these three species from each other. The *ycf4-cemA* could not only distinguish these three species from the other related species, but it could also distinguish *A. longiligulare* from *A. villosum* and *A. villosum* var. *xanthioides*. The NJ trees showed that *accD-psaI* and *ycf4-cemA* were potential markers that could be used to distinguish the three species from their closely related species, but they all had relatively low support rates. All four highly divergent regions could not provide valuable discriminatory power to distinguish these species from each other. Although there are many studies [19,25,43,44,45] using cp genomes to screen regions with high variations which may be used as molecular markers for species authentication, the efficiency and availability of identification remains to be determined.

### 2.6. Phylogenetic Analysis

**Figure 7:**
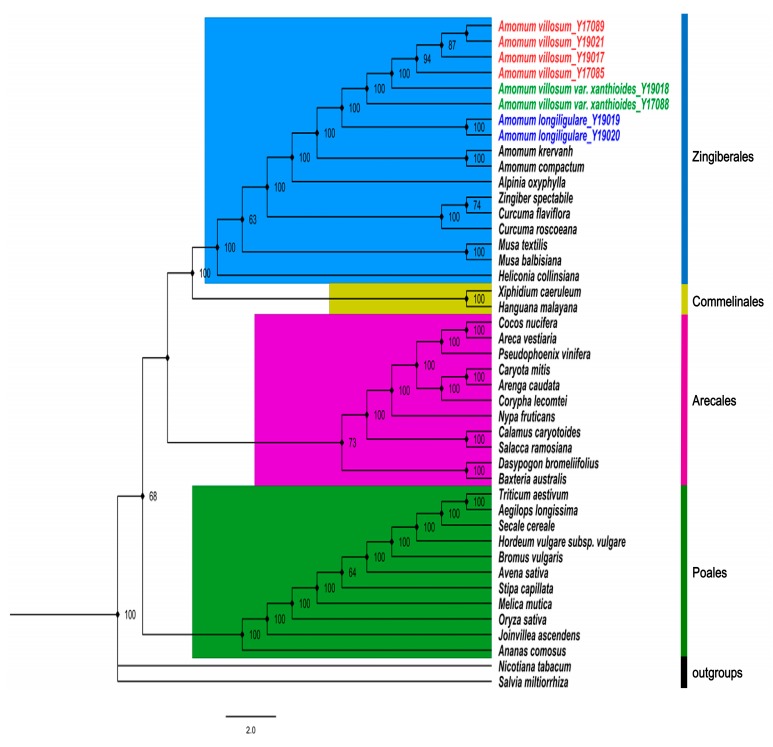
Phylogenetic tree of *A. villosum*, *A. villosum* var. *xanthioides* and *A. longiligulare* inferred by maximum likelihood (ML) analyses based on 64 common protein-coding genes.

To discuss the discriminatory power of cp genomes and determine the phylogenetic positions and evolutionary relationships of *Amomum* species among angiosperm, the maximum likelihood (ML) tree (Figure 7) of 17 representatives from order Zingiberales based on 64 common protein-coding genes, was constructed with *Nicotiana tabacum* and *Salvia miltiorrhiza* as outgroups. On the basis of the topologic structure, each of the four selected families (Zingiberales, Commelinales, Arecales and Poales) was clustered into a monophyletic branch respectively. All five *Amomum* species formed a strongly supported monophyletic clade sister to *Alpinia oxyphylla*, which is sometimes used as an adulterant of amomi fructus. This topological structure thereby hinted at a close relationship between these taxa. In addition, among the three *Amomum* species studied here, branches of four *A. villosum* and two *A. villosum* var. *xanthioides* clustered into a clade, thereby reflecting a closer relationship of these species. Two *A. longiligulare* clustered into a monophyletic branch separately from *A. villosum* and *A. villosum* var. *xanthioides* with high support rates. This showed that common protein-coding genes based on cp genomes could be considered an efficient resource for species authentication in *Amomum*. Results were consistent with traditional phylogenetic theory and provide a valuable basis for the establishment of phylogenetic relationships of species in Zingiberales.

## 3. Materials and Methods

### 3.1. Plant Material, DNA Extraction, and Sequencing

Fresh leaves of 8 individuals of *A. villosum*, *A. villosum* var. *xanthioides* and *A. longiligulare* were collected from their main producing areas. A total of four samples of *A. villosum* were collected respectively, from Yangchun City, Guangdong Province; Nanning City, Guangxi Province; Haikou City, Hainan Province; and Xishuangbanna City, Yunnan Province. Two samples of *A. villosum var. xanthioides* were collected from Xishuangbanna City, Yunnan Province; and two samples of *A. longiligulare* were collected from Haikou City, Hainan Province and Guangzhou City, Guangdong Province. A total of 32 dry leaf samples of eight *Amomum* species (*A. villosum*, *A. villosum* var. *xanthioides*, *A. longiligulare*, *A. chinense*, *A. compactum*, *A. tsaoko*, *A. koenigii*, and *A. maximum*) were collected for verification. The origin information of all samples is listed in Table S4. All samples and vouchers were stored in the herbarium of the Institute of Medicinal Plant Development (IMPLAD), Chinese Academy of Medical Sciences, and Peking Union Medical College (PUMC), and they were identified by Professor Yu-lin Lin. Leaf samples were cleansed by 70% ethanol, and total DNA was extracted using a DNeasy Plant Mini Kit (Qiagen Co., Hilden, Germany) using the standard protocol. DNA concentration and quality were respectively assessed through Nanodrop 2000C spectrophotometry and electrophoresis in 1% (*w*/*v*) agarose gel. The pure DNA from dry leaves was used to perform polymerase chain reaction (PCR) and Sanger sequencing. The pure DNA from fresh leaves was used to construct a shotgun library with an average insert size of 500 bp and carry out next generation sequencing (NGS) by an Illumina HiSeq X 10 platform in accordance with standard protocol.

### 3.2. Cp Genome Assembly

Raw reads obtained from NGS were first checked by FastQC and trimmed using Trimmomatic software [27,46]. The trimmed reads were composed of the data from nuclear and organelle genomes [47]. In order to extract cp-like reads, we mapped the trimmed reads to the reference database, which was constructed by all cp genomes recorded in NCBI, on the basis of their coverage and similarity. The extracted reads were then assembled into contigs by SOAPdenovo [48]. Scaffolds of the cp genome were constructed using SSPACE [49] and gaps were filled using GapCloser [50]. The assembly strategy of raw reads sequenced by Illumina HiSeq X was based on the method of Zhou et al. [19]. The accuracy of assembly of four junctions between the IRs and SSC/LSC regions was verified by PCR amplification and Sanger sequencing using newly designed primers listed in Table S7 and Figure S3.

### 3.3. Cp genome Annotation and Structure Analysis

The complete cp genome was annotated using CPGAVAS [51] and DOGMA [52] with default settings and was checked manually. The tRNA genes were identified by tRNAscan-SE [53]. A BLAST search was used to annotate boundaries of genes, introns/exons and coding regions versus reference sequences. A circular cp genome map was drawn using OGDRAW v1.2 (Organellar Genome DRAW) [54].

The GC content of the cp genome was analyzed using MEGA 6.0 [55]. The distribution of codon usage was investigated using the software CodonW with RSCU ratios. REPuter [56] was used to identify the size and location of repeat sequences, including forward (F), palindromic (P), reverse (R), and complement (C) repeats in the cp genomes. The minimal size for all repeat types was 30 bp, and the two repeat copies had at least 90% similarity. MISA software [57] was used to detect SSRs.

### 3.4. Cp genome Comparisons and Species Authentication

To detect variations within the *Amomum* cp genomes, we compared the cp genomes of *A. villosum*, *A. villosum* var. *xanthioides*, *A. longiligulare* and two other *Amomum* species, namely, *A. kravanh* and *A. compactum*, which were downloaded from GenBank by mVISTA [58]. All cp genome sequences were aligned by MAFFT software [59], and nucleotide diversity of the cp genomes was analyzed through the sliding window by DNA Sequence Polymorphism (DnaSP) [60] software. The step size was set to 200 bp with an 800-bp window length.

In order to evaluate the species discriminatory power for the highly divergent regions, we designed the specific primers for each region (Table S5) and carried out PCR amplification based on some *Amomum* species. PCR amplification was performed in 25-μL reaction mixtures containing 20 ng to ~100 ng of genomic DNA template, 12.5 μL of 2 × EasyTaq PCR SuperMix, and 1 μL of each primer (2.5μM). Purified PCR products were sequenced using the specific primers in the ABI3730XL sequencer. Proofreading and contig assembly of sequencing peak diagrams were performed using CodonCode Aligner 3.7.1 and the sequences were aligned using ClustalW. To evaluate the capability of these sequences to authenticate the studied *Amomum* species, a phylogenetic tree was constructed using the neighbor-joining algorithm (NJ tree), and bootstrap tests were conducted using 1000 resamples to assess the confidence of the phylogenetic relationships using MEGA 6.0.

### 3.5. Phylogenetic Analyses

To discuss the discriminatory power of the cp genomes and reveal the phylogenetic position of *Amomum* species, we downloaded 35 complete cp genome sequences from NCBI’s Organelle Genome and Nucleotide Resources database (Table S8). The sequences of 64 common protein-coding genes (*atpA*, *atpB*, *atpE*, *atpF*, *atpH*, *atpI*, *ccsA*, *cemA*, *clpP*, *matK*, *ndhA*, *ndhB*, *ndhC*, *ndhD*, *ndhE*, *ndhF*, *ndhG*, *ndhH*, *ndhI*, *ndhJ*, *ndhK*, *petA*, *petG*, *petL*, *petN*, *psaA*, *psaB*, *psaC*, *psaI*, *psaJ*, *psbA*, *psbB*, *psbC*, *psbD*, *psbE*, *psbF*, *psbH*, *psbI*, *psbJ*, *psbK*, *psbL*, *psbM*, *psbN*, *psbT*, *rbcL*, *rpl14*, *rpl2*, *rpl22*, *rpl32*, *rpl33*, *rpl36*, *rpoA*, *rpoB*, *rpoC1*, *rps11*, *rps16*, *rps19*, *rps2*, *rps3*, *rps4*, *rps7*, *rps8*, *ycf3*, and *ycf4*) shared in these species were extracted and aligned separately using MAFFT v7 [61], and the alignment was manually verified. Phylogenetic trees were reconstructed based on the 64 concatenated protein-coding gene sequences by ML methods. ML analysis was conducted with a bootstrap of 1000 repetitions based on the GTR+F+R3 nucleotide substitution model using IQ-TREE software [62]. This adopted best-fit model was also determined by IQ-TREE. *N. tabacum* and *S. miltiorrhiza* were set as outgroups.

## 4. Conclusions

This is the first study to sequence and determine the complete cp genomes of eight individual species of *A. villosum*, *A. villosum* var. *xanthioides* and *A. longiligulare* via high-throughput sequencing, which were collected from the different main producing areas in China. First of all, we obtained and compared eight complete cp genomes, and we found that the cp genomes of the same species from different producing areas showed little difference. Then, five divergent regions (*atpH-atpI*, *trnD-trnY*, *accD-psaI*, *ycf4-cemA* and *trnI-ycf2*), which were detected by comparative analyses using Sanger sequencing based on 32 samples, proved to not be useful as effective molecular markers to identify species in *Amomum*. Further, phylogenetic analyses revealed that cp genomes could be used to identify *Amomum* species. The availability of cp genome sequences is expected to improve species identification and phylogenetic analyses in *Amomum*.

## Figures and Tables

**Table 1:** Comparisons among the cp genome characteristics of *A. villosum*, *A. villosum* var. *xanthioides* and *A. longiligulare*.

Type	*A. Villosum*	*A. Villosum* var. *Xanthioides*	*A. Longiligulare*
Accession Number	MH161416 MH161418 MN067431 MN067432	MH161417 MN067433	MN067434 MN067435
Total Length (bp)	164,068–164,069	163,981–163,985	163,608
LSC Length (bp)	88,797–88,798	88,720–88,857	88,680
IR Length (bp)	29,959	29,886–29,948	29,820
SSC Length (bp)	135,352–135,353	15,352–15,369	15,288
CDS Length (bp)	83,190	83,178–83,196	83,160
Total GC content	36.0%	36.0%	36.1%
GC content of LSC	33.7%	33.7%	33.7%
GC content of IRa	41.1%	41.1%	41.1%
GC content of IRb	41.1%	41.1%	41.1%
GC content of SSC	30.0%	30.0%	30.1%
AT content at 1st position	55.4%	55.4%	55.4%
AT content at 2nd position	62.6%	62.6%	62.6%
AT content at 3rd position	71.2%	71.2%	71.2%

**Table 2:** Gene contents in the cp genomes of *A. villosum*, *A. villosum* var. *xanthioides* and *A. longiligulare*.

Group of Genes	Gene Names	Number of Genes
Photosystem I	*psaA*, *psaB*, *psaC*, *psaI*, *psaJ*	5
Photosystem II	*psbA*, *psbB*, *psbC*, *psbD*, *psbE*, *psbF*, *psbH*, *psbI*, *psbJ*, *psbK*, *psbL*, *psbM*, *psbN*, *psbT*, *psbZ*	15
Cytochrome b/f complex	*petA*, petB *, petD *, *petG*, *petL*, *petN*	6
ATP synthase	*atpA*, *atpB*, *atpE*, atpF *, *atpH*, *atpI*	6
NADH dehydrogenase	ndhA *, *ndhB*(×2) *, *ndhC*, *ndhD*, *ndhE*, *ndhF*, *ndhG*, *ndhH*, *ndhI*, *ndhJ*, *ndhK*	12
RubisCO large subunit	*rbcL*	1
RNA polymerase	*rpoA*, *rpoB*, rpoC1 *, *rpoC2*	4
Ribosomal proteins (SSU)	*rps2*, *rps3*, *rps4*, *rps7*(×2), *rps8*, *rps11*, *rps12*(×2) **, *rps14*, *rps15*, rps16 *, *rps18*, *rps19*(×2)	15
Ribosomal proteins (LSU)	*rpl2*(×2) *, *rpl14*, rpl16 *, *rpl20*, *rpl22*, *rpl23*(×2), *rpl32*, *rpl33*, *rpl36*	11
Proteins of unknown function	*ycf1*(×2), *ycf2*(×2), ycf3 **, *ycf4*	6
Transfer RNAs	38 *tRNA*s (8 in the IRs(×2), 6 contain one intron)	38
Ribosomal RNAs	*rrn4.5*(×2), *rrn5*(×2), *rrn16*(×2), *rrn23*(×2)	8
Other genes	*accD*, clpP **, *matK*, *ccsA*, *cemA*, *infA*	6

(×2) indicates the gene sequence is repeated twice. * indicates genes containing one intron; while ** indicates gene containing two introns. The *rps12* gene is a trans-spliced gene.

## Supplementary Materials

Supplementary materials can be found at https://www.mdpi.com/1422-0067/20/16/4040/s1.

## Abbreviations

LSC	Large single copy
SSC	Small single copy
IR	Inverted repeat
ML	Maximum likelihood
SSR	Simple sequence repeats
ATP	Adenosine triphosphate
NADH	Nicotinamide adenine dinucleotide
