# Supplementary material for: Comparison and Phylogenetic Analysis of Chloroplast Genomes of Three Medicinal and Edible Amomum Species
Source: Int J Mol Sci. 2019 Aug 19;20(16):4040. doi: 10.3390/ijms20164040 (PMC6720276; doi:10.3390/ijms20164040)
Supplement: Supplementary file 1 [file ijms-20-04040-s001.pdf]

Comparison and Phylogenetic Analysis of Chloroplast Genomes of Three Medicinal and Edible *Amomum* Species

Yingxian Cui [1,2], Xinlian Chen [1,2], Liping Nie [1,2], Wei Sun [3], Haoyu Hu [3], Yulin Lin [1,2], Haitao Li [4], Xilong Zheng [5], Jingyuan Song [1,2] and Hui Yao [1,2,*]

1. Key Lab of Chinese Medicine Resources Conservation, State Administration of Traditional Chinese Medicine of the People's Republic of China, Institute of Medicinal Plant Development, Chinese Academy of Medical Sciences & Peking Union Medical College, Beijing 100193, China
2. Engineering Research Center of Chinese Medicine Resources, Ministry of Education, Beijing 100193, China
3. Institute of Chinese Materia Medica, China Academy of Chinese Medical Sciences, Beijing 100700, China
4. Yunnan Branch, Institute of Medicinal Plant Development, Chinese Academy of Medical Sciences & Peking Union Medical College, Jinghong 666100, China
5. Hainan Branch, Institute of Medicinal Plant Development, Chinese Academy of Medical Sciences & Peking Union Medical College, Wanning 571533, China

* Correspondence: scauyaoh@sina.com; Tel.: +86-10-57833194

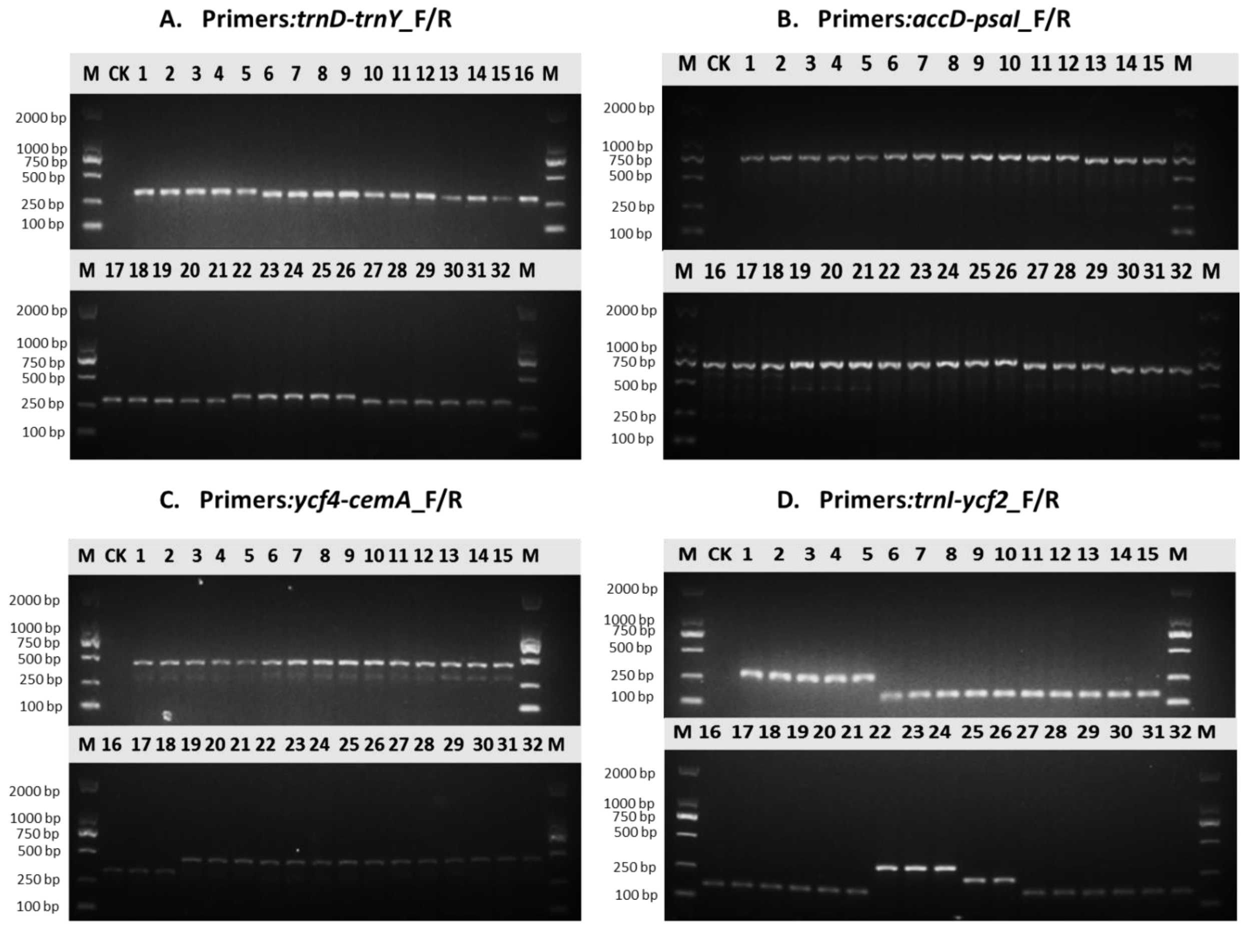

Figure S1. The PCR amplification results of the highly divergent regions, including *trnD-trnY*, *accD-psaI*, *ycf4-cemA*, and *trnI-ycf2*. M: Maker DL 2000; CK: Control Check; Sample order: 1-5: Y19005MT01-05 (*A. villosum*); 6-10: Y19006MT01-05 (*A. longiligulare*); 11-12: Y19007MT01-02 (*A. longiligulare*); 13-15: Y19008MT01-03 (*A. chinense*); 16-18: Y19009MT01-03 (*A. ompactum*); 19-21: Y19010MT01 -03 (*A. saoko*); 22-24: Y19011MT01-03 (*A. villosum*); 25-26: Y19012MT01-02 (*A. villosum* var. *xanthioides*); 27-29: Y19013MT01-03 (*A. koenigii*); 30-32: Y19014MT01-03 (*A. maximum*).

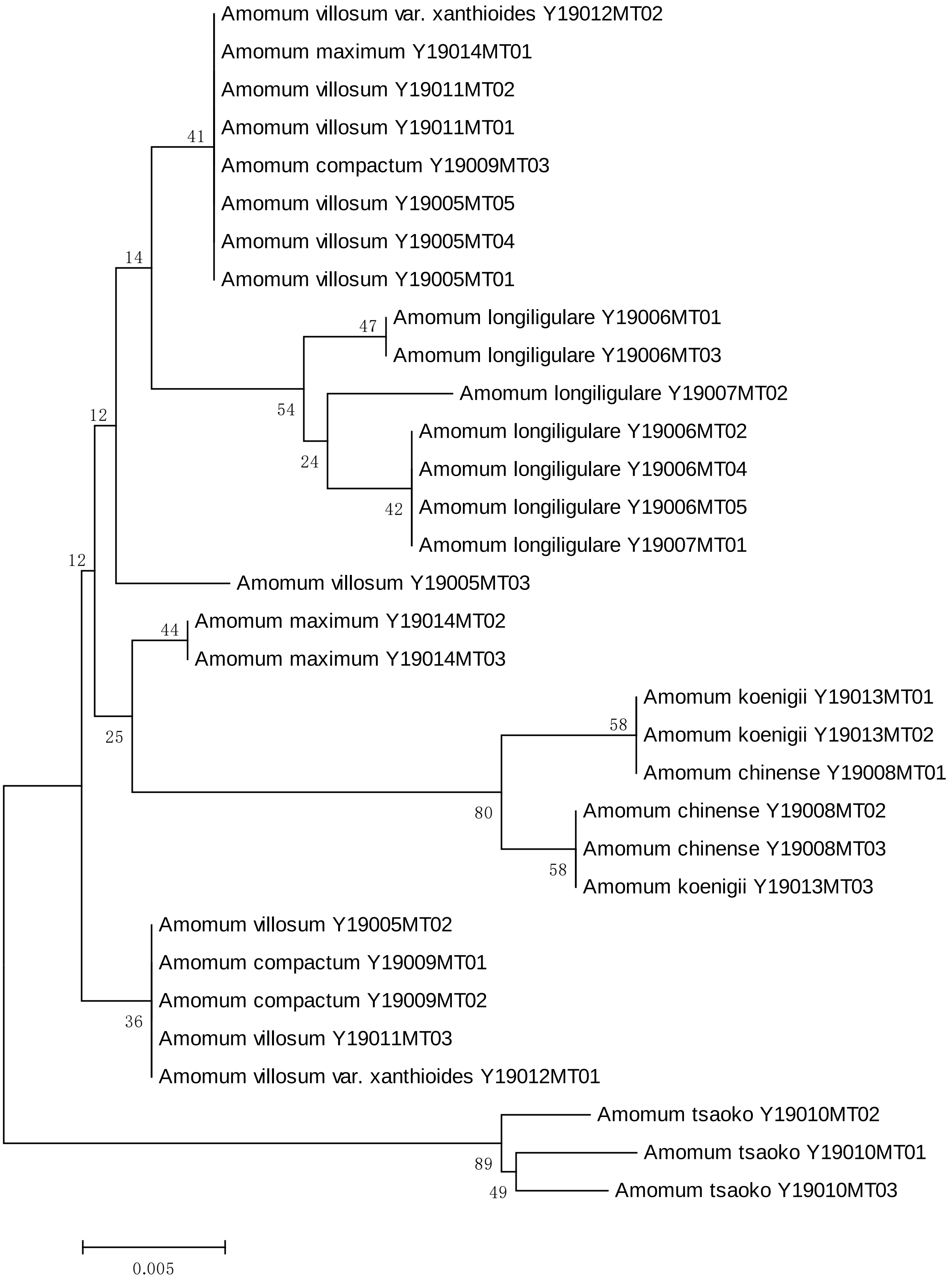
Amomum villosum var. xanthioides Y19012MT02
Amomum maximum Y19014MT01
Amomum villosum Y19011MT02
Amomum villosum Y19011MT01
Amomum compactum Y19009MT03
Amomum villosum Y19005MT05
Amomum villosum Y19005MT04
Amomum villosum Y19005MT01
Amomum longiligulare Y19006MT01
Amomum longiligulare Y19006MT03
Amomum longiligulare Y19007MT02
Amomum longiligulare Y19006MT02
Amomum longiligulare Y19006MT04
Amomum longiligulare Y19006MT05
Amomum longiligulare Y19007MT01
Amomum villosum Y19005MT03
Amomum maximum Y19014MT02
Amomum maximum Y19014MT03
Amomum koenigii Y19013MT01
Amomum koenigii Y19013MT02
Amomum chinense Y19008MT01
Amomum chinense Y19008MT02
Amomum chinense Y19008MT03
Amomum koenigii Y19013MT03
Amomum villosum Y19005MT02
Amomum compactum Y19009MT01
Amomum compactum Y19009MT02
Amomum villosum Y19011MT03
Amomum villosum var. xanthioides Y19012MT01
Amomum tsaoko Y19010MT02
Amomum tsaoko Y19010MT01
Amomum tsaoko Y19010MT03
41
14
47
54
12
24
42
12
44
25
58
80
58
36
89
49
0.005

A. *trnD-trnY*

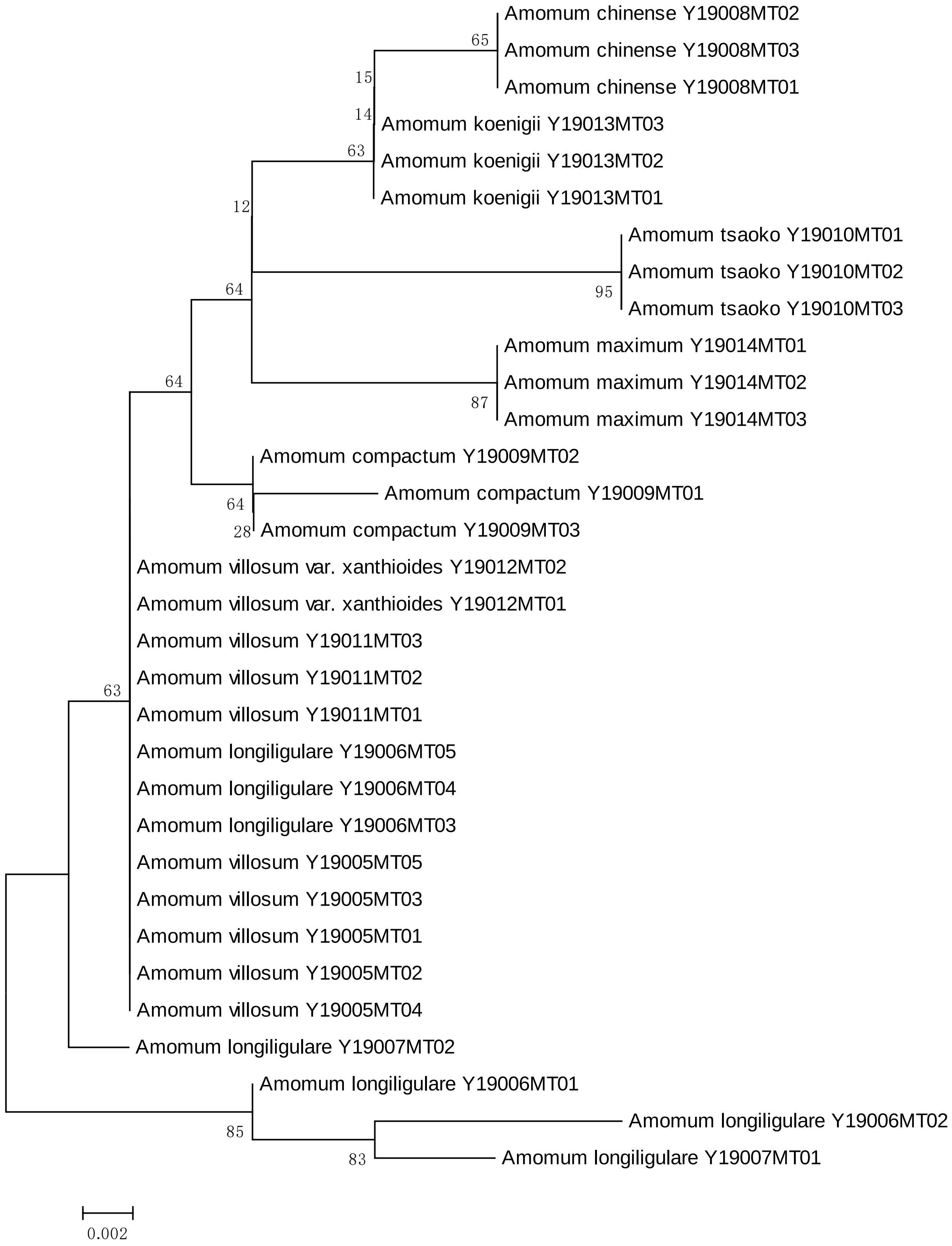
Amomum chinense Y19008MT02
Amomum chinense Y19008MT03
Amomum chinense Y19008MT01
Amomum koenigii Y19013MT03
Amomum koenigii Y19013MT02
Amomum koenigii Y19013MT01
Amomum tsaoko Y19010MT01
Amomum tsaoko Y19010MT02
Amomum tsaoko Y19010MT03
Amomum maximum Y19014MT01
Amomum maximum Y19014MT02
Amomum maximum Y19014MT03
Amomum compactum Y19009MT02
Amomum compactum Y19009MT01
Amomum compactum Y19009MT03
Amomum villosum var. xanthioides Y19012MT02
Amomum villosum var. xanthioides Y19012MT01
Amomum villosum Y19011MT03
Amomum villosum Y19011MT02
Amomum villosum Y19011MT01
Amomum longiligulare Y19006MT05
Amomum longiligulare Y19006MT04
Amomum longiligulare Y19006MT03
Amomum villosum Y19005MT05
Amomum villosum Y19005MT03
Amomum villosum Y19005MT01
Amomum villosum Y19005MT02
Amomum villosum Y19005MT04
Amomum longiligulare Y19007MT02
Amomum longiligulare Y19006MT01
Amomum longiligulare Y19006MT02
Amomum longiligulare Y19007MT01
65
15
14
63
12
64
95
64
87
64
28
63
85
83
0.002

B. *accD- psaI*

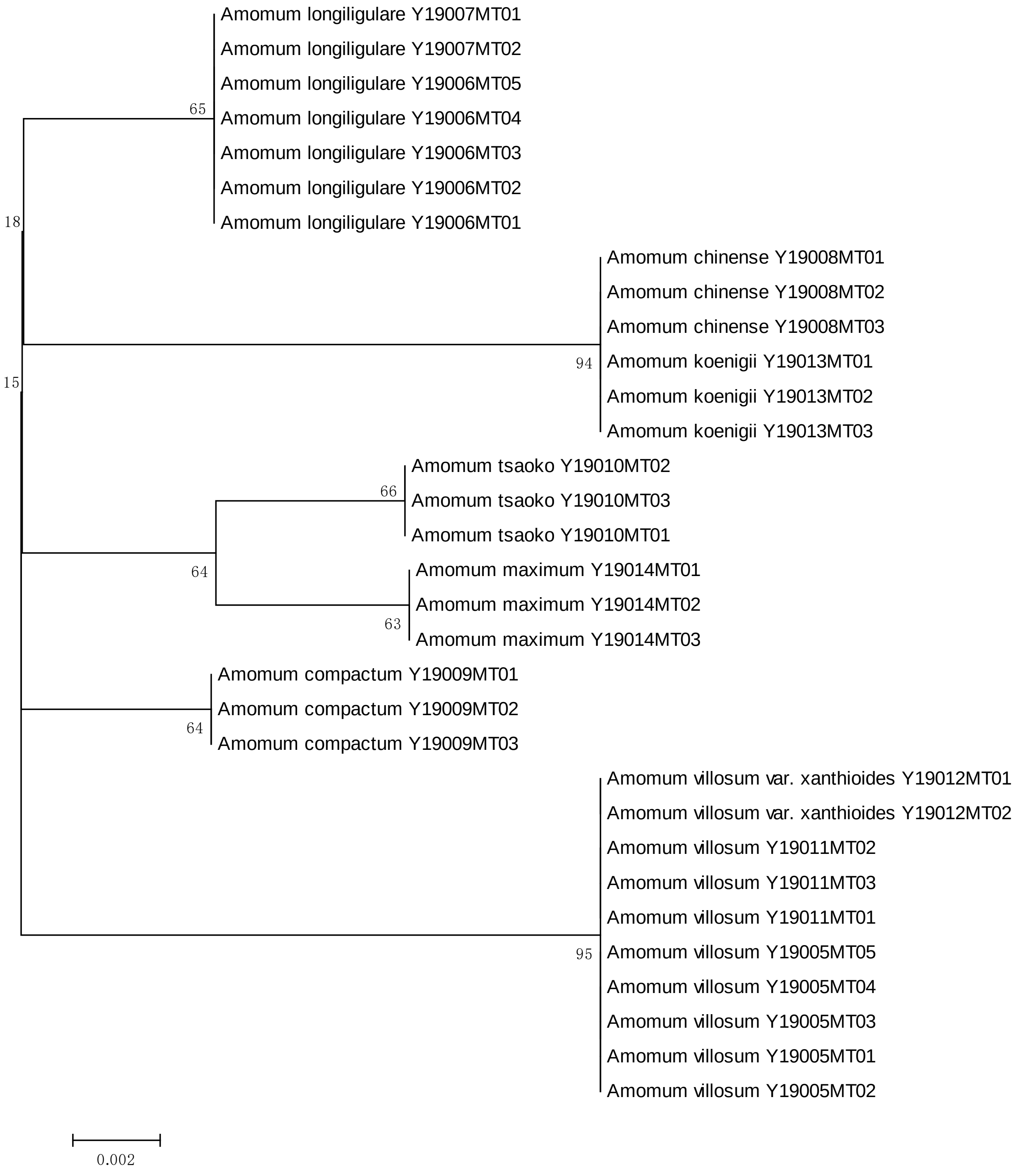
Amomum longiligulare Y19007MT01
Amomum longiligulare Y19007MT02
Amomum longiligulare Y19006MT05
Amomum longiligulare Y19006MT04
Amomum longiligulare Y19006MT03
Amomum longiligulare Y19006MT02
Amomum longiligulare Y19006MT01
65
18
Amomum chinense Y19008MT01
Amomum chinense Y19008MT02
Amomum chinense Y19008MT03
Amomum koenigii Y19013MT01
Amomum koenigii Y19013MT02
Amomum koenigii Y19013MT03
94
15
Amomum tsaoko Y19010MT02
Amomum tsaoko Y19010MT03
Amomum tsaoko Y19010MT01
66
64
Amomum maximum Y19014MT01
Amomum maximum Y19014MT02
Amomum maximum Y19014MT03
63
Amomum compactum Y19009MT01
Amomum compactum Y19009MT02
Amomum compactum Y19009MT03
64
Amomum villosum var. xanthioides Y19012MT01
Amomum villosum var. xanthioides Y19012MT02
Amomum villosum Y19011MT02
Amomum villosum Y19011MT03
Amomum villosum Y19011MT01
Amomum villosum Y19005MT05
Amomum villosum Y19005MT04
Amomum villosum Y19005MT03
Amomum villosum Y19005MT01
Amomum villosum Y19005MT02
95
0.002

C. *ycf4-cemA*

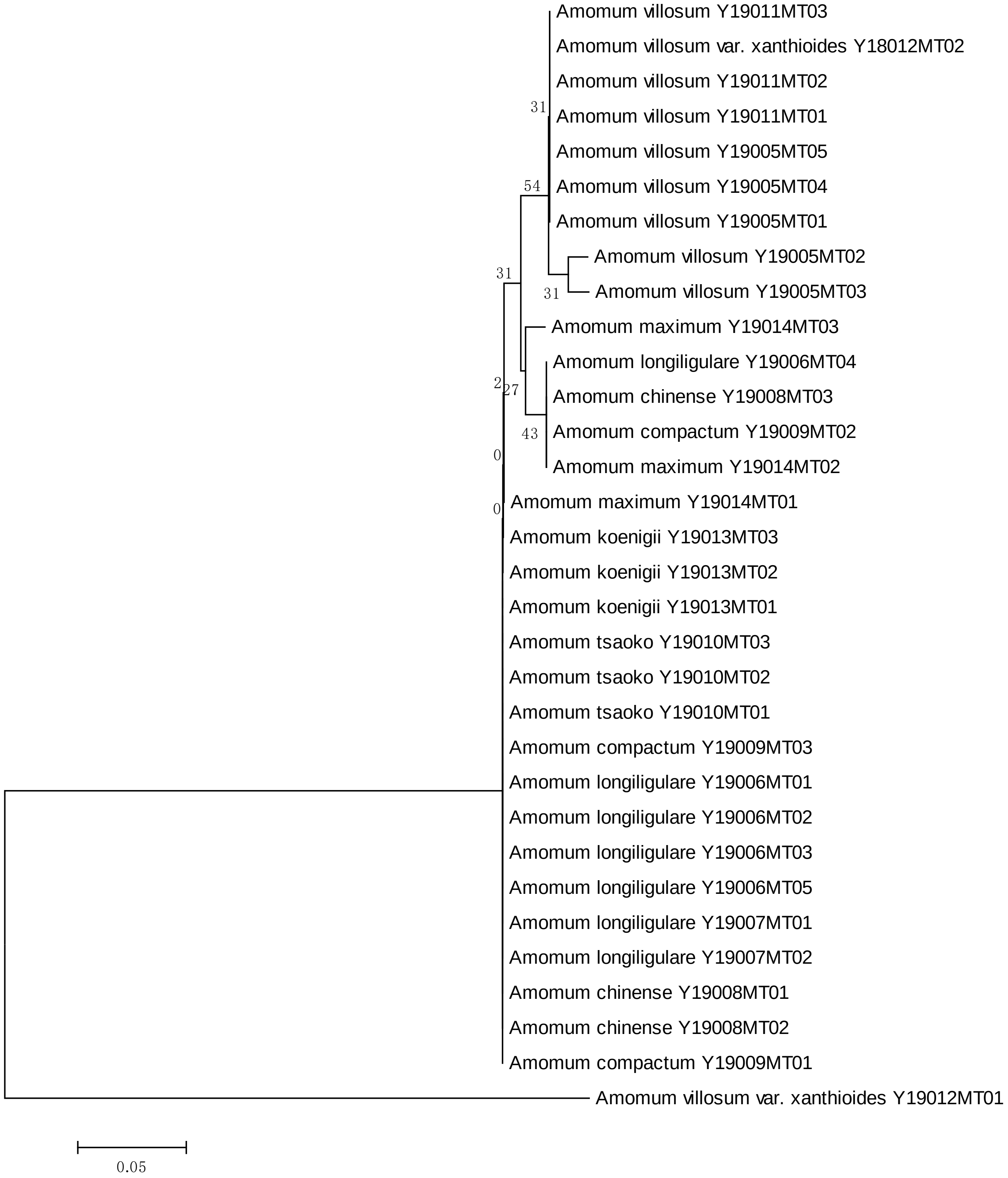

D. *trnI-ycf2*

Figure S2. The NJ tree of *Amomum* species constructed with the highly divergent regions, including *trnD-trnY, accD-psaI, ycf4-cemA,* and *trnI-ycf2*. The bootstrap score (1000 replicates) are shown for each branch.

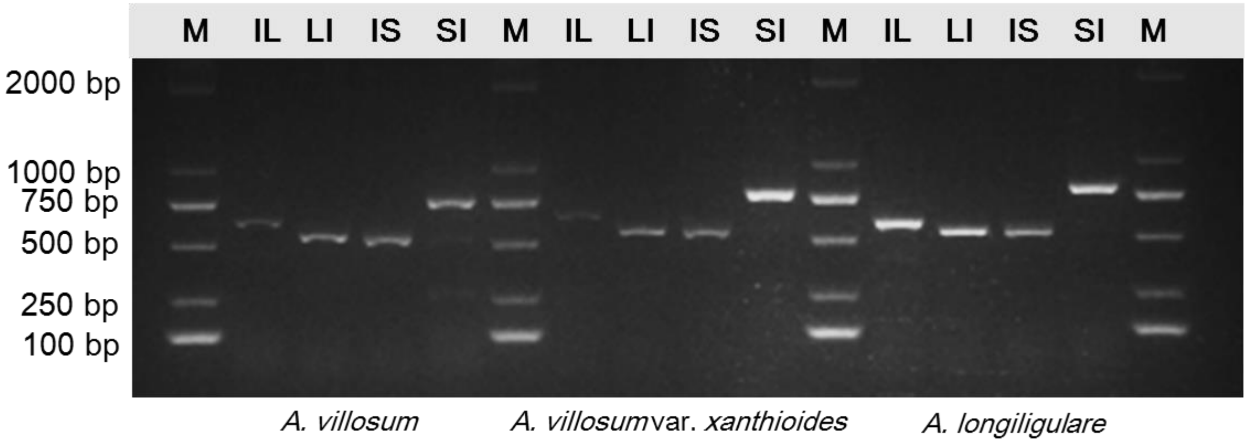

Figure S3. The PCR amplification results of four junctions between the IRs and SSC/LSC regions of of *A. villosum*, *A. villosum* var. *xanthioides* and *A. longiligulare*.. IL: IRb-LSC; LI: LSC-IRa; IS: IRa-SSC; SI: SSC-IRb.

Table S1. Genes with introns in the chloroplast genomes of *A. villosum, A. villosum* var. *xanthioides* and *A. longiligulare.* as well as the lengths of the exons and introns.

Species	Gene	Location	Exon I (bp)	Intron I (bp)	Exon II (bp)	Intron II (bp)	Exon III (bp)
A. villosum	*atpF*	LSC	144	799	411		
	clpP	LSC	69	844	296	630	253
	ndhA	SSC	556	1050	539		
	ndhB	IR	777	700	756		
	petB	LSC	6	872	642		
	petD	LSC	8	749	475		
	rpl16	LSC	9	1049	402		
	rpl2	IR	391	662	431		
	rpoC1	LSC	432	723	1638		
	rps7	IR	231	551	27		
	*rps12**	LSC	114	-	231	553	27
	rps16	LSC	40	727	218		
	trnA-UGC	IR	38	801	35		
	trnG-UCC	LSC	23	702	47		
	trnI-GAU	IR	42	935	35		
	trnK-UUU	LSC	37	2688	35		
	trnL-UAA	LSC	35	532	50		
	trnV-UAC	LSC	38	604	37		
	trnV-GAC	IR	27	551	231		
	ycf3	LSC	127	715	230	777	153
A. villosum var. *xanthioides*	*atpF*	LSC	144	799	411		
	clpP	LSC	69	842	296	640	253
	ndhA	SSC	556	1045	539		
	ndhB	IR	777	700	756		
	petB	LSC	6	870	642		
	petD	LSC	8	749	475		
	rpl16	LSC	9	1046	402		
	rpl2	IR	391	662	431		
	rpoC1	LSC	432	727	1638		
	rps7	IR	231	551	27		
	*rps12**	LSC	114	-	231	553	27
	rps16	LSC	40	730	218		
	trnA-UGC	IR	38	801	35		
	trnG-UCC	LSC	23	703	47		
	trnI-GAU	IR	42	935	35		
	trnK-UUU	LSC	37	2688	35		
	trnL-UAA	LSC	35	533	50		
	trnV-UAC	LSC	38	604	37		
	trnV-GAC	IR	27	551	231		
	ycf3	LSC	127	714	230	777	153
A. longiligulare	*atpF*	LSC	144	799	411		
	clpP	LSC	69	844	296	630	253
	ndhA	SSC	556	1049	539		

ndhB	IR	777	700	756		
petB	LSC	6	872	642		
petD	LSC	8	749	475		
rpl16	LSC	9	1049	402		
rpl2	IR	391	662	431		
rpoC1	LSC	432	723	1638		
rps7	IR	231	551	27		
*rps12**	LSC	114	-	231	553	27
rps16	LSC	40	727	218		
trnA-UGC	IR	38	801	35		
trnG-UCC	LSC	23	702	47		
trnI-GAU	IR	42	935	35		
trnK-UUU	LSC	37	2688	35		
trnL-UAA	LSC	35	532	50		
trnV-UAC	LSC	38	604	37		
trnV-GAC	IR	27	551	231		
ycf3	LSC	127	715	230	777	153

* The *rps12* gene is a trans-spliced gene with the 5’ end located in the LSC region and the duplicated 3’ ends in the IR regions.

Table S2. Codon-anticodon recognition pattern and codon usage for the chloroplast genomes of *A. villosum*, *A. villosum* var. *xanthioides* and *A. longiligulare*.

Species	Amino acid	Codon	No.	RSCU	tRNA	Amino acid	Codon	No.	RSCU	tRNA
A. villosum	Phe	UUU	1010	1.29		Tyr	UAU	842	1.58	
	Phe	UUC	558	0.71	trnF-GAA	Tyr	UAC	227	0.42	trnY-GUA
	Leu	UUA	913	1.92	trnL-UAA	Stop	UAA	49	1.69	
	Leu	UUG	588	1.24	trnL-CAA	Stop	UAG	22	0.76	
	Leu	CUU	598	1.26		His	CAU	533	1.6	
	Leu	CUC	198	0.42		His	CAC	135	0.4	trnH-GUG
	Leu	CUA	399	0.84	trnL-UAG	Gln	CAA	735	1.53	trnQ-UUG
	Leu	CUG	157	0.33		Gln	CAG	224	0.47	
	Ile	AUU	1191	1.47		Asn	AAU	1055	1.54	
	Ile	AUC	448	0.55	trnI-GAU	Asn	AAC	314	0.46	trnN-GUU
	Ile	AUA	786	0.97	trnI-CAU	Lys	AAA	1183	1.48	trnK-UUU
	Met	AUG	645	1	trn(f)M-CAU	Lys	AAG	415	0.52	
	Val	GUU	534	1.44		Asp	GAU	914	1.64	
	Val	GUC	172	0.46	trnV-GAC	Asp	GAC	198	0.36	trnD-GUC
	Val	GUA	573	1.55	trnV-UAC	Glu	GAA	1161	1.51	trnE-UUC
	Val	GUG	204	0.55		Glu	GAG	376	0.49	
	Ser	UCU	638	1.76		Cys	UGU	246	1.55	
	Ser	UCC	350	0.97	trnS-GGA	Cys	UGC	71	0.45	trnC-GCA
	Ser	UCA	434	1.2	trnS-UGA	Stop	UGA	16	0.55	
	Ser	UCG	195	0.54		Trp	UGG	477	1	trnW-CCA
	Pro	CCU	460	1.64		Arg	CGU	371	1.33	trnR-ACG
	Pro	CCC	208	0.74		Arg	CGC	93	0.33	
	Pro	CCA	332	1.18	trnP-UGG	Arg	CGA	361	1.3	
	Pro	CCG	124	0.44		Arg	CGG	119	0.43	
	Thr	ACU	547	1.55		Arg	AGA	446	1.23	trnR-UCU
	Thr	ACC	248	0.7	trnT-GGU	Arg	AGG	109	0.3	
	Thr	ACA	452	1.28	trnT-UGU	Ser	AGU	549	1.97	
	Thr	ACG	168	0.47		Ser	AGC	179	0.64	trnS-GCU
	Ala	GCU	633	1.81		Gly	GGU	618	1.39	
	Ala	GCC	204	0.58		Gly	GGC	142	0.32	trnG-GCC
	Ala	GCA	443	1.27	trnA-UGC	Gly	GGA	730	1.64	trnG-UCC
	Ala	GCG	118	0.34		Gly	GGG	292	0.66	
A. villosum var. *xanthioides*	Phe	UUU	1013	1.29		Tyr	UAU	847	1.58	
	Phe	UUC	559	0.71	trnF-GAA	Tyr	UAC	225	0.42	trnY-GUA
	Leu	UUA	915	1.93	trnL-UAA	Stop	UAA	49	1.69	
	Leu	UUG	583	1.23	trnL-CAA	Stop	UAG	22	0.76	
	Leu	CUU	596	1.26		His	CAU	530	1.59	
	Leu	CUC	196	0.41		His	CAC	135	0.41	trnH-GUG
	Leu	CUA	397	0.84	trnL-UAG	Gln	CAA	736	1.53	trnQ-UUG
	Leu	CUG	157	0.33		Gln	CAG	223	0.47	
	Ile	AUU	1193	1.47		Asn	AAU	1055	1.54	
	Ile	AUC	449	0.55	trnI-GAU	Asn	AAC	313	0.46	trnN-GUU
	Ile	AUA	786	0.97	trnI-CAU	Lys	AAA	1183	1.48	trnK-UUU

	Met	AUG	645	1	trn(f)M-CAU	Lys	AAG	414	0.52	
	Val	GUU	535	1.44		Asp	GAU	915	1.64	
	Val	GUC	171	0.46	trnV-GAC	Asp	GAC	200	0.36	trnD-GUC
	Val	GUA	575	1.55	trnV-UAC	Glu	GAA	1160	1.51	trnE-UUC
	Val	GUG	203	0.55		Glu	GAG	378	0.49	
	Ser	UCU	634	1.75		Cys	UGU	246	1.55	
	Ser	UCC	349	0.97	trnS-GGA	Cys	UGC	71	0.45	trnC-GCA
	Ser	UCA	436	1.21	trnS-UGA	Stop	UGA	16	0.55	
	Ser	UCG	195	0.54		Trp	UGG	477	1	trnW-CCA
	Pro	CCU	459	1.63		Arg	CGU	371	1.33	trnR-ACG
	Pro	CCC	209	0.74		Arg	CGC	93	0.33	
	Pro	CCA	332	1.18	trnP-UGG	Arg	CGA	359	1.29	
	Pro	CCG	125	0.44		Arg	CGG	119	0.43	
	Thr	ACU	546	1.55		Arg	AGA	447	1.24	trnR-UCU
	Thr	ACC	247	0.7	trnT-GGU	Arg	AGG	108	0.3	
	Thr	ACA	452	1.28	trnT-UGU	Ser	AGU	548	1.97	
	Thr	ACG	168	0.48		Ser	AGC	180	0.65	trnS-GCU
	Ala	GCU	634	1.81		Gly	GGU	620	1.39	
	Ala	GCC	204	0.58		Gly	GGC	140	0.31	trnG-GCC
	Ala	GCA	442	1.26	trnA-UGC	Gly	GGA	730	1.64	trnG-UCC
	Ala	GCG	119	0.34		Gly	GGG	292	0.66	
A. longiligulare	Phe	UUU	1010	1.29		Tyr	UAU	842	1.58	
	Phe	UUC	558	0.71	trnF-GAA	Tyr	UAC	227	0.42	trnY-GUA
	Leu	UUA	913	1.92	trnL-UAA	Stop	UAA	49	1.69	
	Leu	UUG	588	1.24	trnL-CAA	Stop	UAG	22	0.76	
	Leu	CUU	598	1.26		His	CAU	533	1.6	
	Leu	CUC	198	0.42		His	CAC	135	0.4	trnH-GUG
	Leu	CUA	399	0.84	trnL-UAG	Gln	CAA	735	1.53	trnQ-UUG
	Leu	CUG	157	0.33		Gln	CAG	224	0.47	
	Ile	AUU	1191	1.47		Asn	AAU	1056	1.54	
	Ile	AUC	448	0.55	trnI-GAU	Asn	AAC	314	0.46	trnN-GUU
	Ile	AUA	786	0.97	trnI-CAU	Lys	AAA	1183	1.48	trnK-UUU
	Met	AUG	645	1	trn(f)M-CAU	Lys	AAG	414	0.52	
	Val	GUU	534	1.44		Asp	GAU	914	1.64	
	Val	GUC	172	0.46	trnV-GAC	Asp	GAC	198	0.36	trnD-GUC
	Val	GUA	573	1.55	trnV-UAC	Glu	GAA	1161	1.51	trnE-UUC
	Val	GUG	204	0.55		Glu	GAG	376	0.49	
	Ser	UCU	638	1.76		Cys	UGU	246	1.55	
	Ser	UCC	350	0.97	trnS-GGA	Cys	UGC	71	0.45	trnC-GCA
	Ser	UCA	434	1.2	trnS-UGA	Stop	UGA	16	0.55	
	Ser	UCG	195	0.54		Trp	UGG	477	1	trnW-CCA
	Pro	CCU	460	1.64		Arg	CGU	371	1.33	trnR-ACG
	Pro	CCC	208	0.74		Arg	CGC	93	0.33	
	Pro	CCA	332	1.18	trnP-UGG	Arg	CGA	361	1.3	
	Pro	CCG	124	0.44		Arg	CGG	119	0.43	

Thr	ACU	547	1.55		Arg	AGA	446	1.23	trnR-UCU
Thr	ACC	248	0.7	trnT-GGU	Arg	AGG	109	0.3	
Thr	ACA	452	1.28	trnT-UGU	Ser	AGU	549	1.97	
Thr	ACG	168	0.47		Ser	AGC	179	0.64	trnS-GCU
Ala	GCU	633	1.81		Gly	GGU	618	1.39	
Ala	GCC	204	0.58		Gly	GGC	142	0.32	trnG-GCC
Ala	GCA	443	1.27	trnA-UGC	Gly	GGA	730	1.64	trnG-UCC
Ala	GCG	118	0.34		Gly	GGG	292	0.66	

RSCU: Relative Synonymous Codon Usage.

Table S3. Simple sequence repeats (SSRs) in the complete chloroplast genome of *A. villosum*, *A. villosum* var. *xanthioides* and *A. longiligulare*.

Species	SSR nr.	SSR type	SSR	size	start	end
A. villosum	1	p1	(A)10	10	16	25
	2	p3	(GCT)4	12	722	733
	3	p1	(T)10	10	1716	1725
	4	p2	(AT)5	10	2690	2699
	5	p2	(AT)5	10	3471	3480
	6	p1	(A)10	10	3724	3733
	7	p1	(T)11	11	4014	4024
	8	p1	(A)10	10	4368	4377
	9	p2	(TA)5	10	4965	4974
	10	p2	(TA)6	12	4989	5000
	11	p1	(T)10	10	5021	5030
	12	p1	(A)15	15	5378	5392
	13	p1	(A)10	10	5732	5741
	14	p1	(T)11	11	5743	5753
	15	p2	(TA)5	10	6248	6257
	16	p1	(A)10	10	6350	6359
	17	p2	(TA)8	16	6717	6732
	18	p1	(A)10	10	7316	7325
	19	p1	(A)10	10	7574	7583
	20	p2	(AT)5	10	7635	7644
	21	p1	(A)11	11	7812	7822
	22	p5	(TTAAA)3	15	9362	9376
	23	p1	(T)10	10	10075	10084
	24	p1	(A)10	10	10754	10763
	25	p4	(TTAT)3	12	13057	13068
	26	p1	(T)10	10	13236	13245
	27	p1	(T)10	10	13248	13257
	28	p1	(A)11	11	13290	13300
	29	p1	(T)10	10	13972	13981
	30	p1	(T)10	10	13987	13996
	31	p4	(TTTA)3	12	14001	14012
	32	p4	(AAAG)3	12	14210	14221
	33	p1	(A)10	10	14971	14980
	34	p6	(AATATA)3	18	15097	15114
	35	p5	(ATATA)3	15	15139	15153
	36	p2	(AT)7	14	15253	15266
	37	p1	(T)10	10	16780	16789
	38	p1	(T)10	10	17598	17607
	39	p1	(T)10	10	19708	19717
	40	p1	(T)12	12	19813	19824
	41	p1	(A)10	10	19956	19965
	42	p2	(AT)5	10	21184	21193
	43	p1	(T)12	12	24249	24260
	44	p2	(TA)5	10	28710	28719

45	p1	(A)13	13	28917	28929
46	p1	(A)12	12	29126	29137
47	p1	(T)12	12	29532	29543
48	p1	(T)11	11	29631	29641
49	p1	(A)10	10	29643	29652
50	p1	(A)11	11	30973	30983
51	p1	(A)10	10	33234	33243
52	p1	(T)11	11	33823	33833
53	p2	(AT)5	10	34378	34387
54	p3	(TCT)4	12	35146	35157
55	p1	(T)11	11	35188	35198
56	p1	(T)12	12	35427	35438
57	p2	(TA)7	14	38686	38699
58	p1	(T)10	10	39122	39131
59	p1	(A)11	11	39357	39367
60	p1	(A)10	10	39571	39580
61	p1	(A)11	11	47271	47281
62	p1	(A)13	13	47535	47547
63	p5	(AATCA)3	15	48173	48187
64	p1	(T)10	10	49845	49854
65	p2	(TA)6	12	49910	49921
66	p2	(TA)5	10	49939	49948
67	p1	(A)13	13	50299	50311
68	p2	(AT)8	16	50461	50476
69	p4	(CAAA)3	12	50575	50586
70	p1	(T)10	10	51890	51899
71	p1	(T)11	11	52777	52787
72	p1	(T)10	10	53524	53533
73	p3	(ATA)5	15	54413	54427
74	p4	(TAAT)3	12	54463	54474
75	p1	(A)10	10	54637	54646
76	p1	(A)10	10	55412	55421
77	p4	(ATTT)3	12	56396	56407
78	p1	(T)11	11	58876	58886
79	p1	(T)11	11	59366	59376
80	p1	(A)12	12	61016	61027
81	p1	(T)11	11	61689	61699
82	p1	(A)10	10	63298	63307
83	p2	(AT)7	14	63343	63356
84	c	(TG)5(T)14	24	63434	63457
85	p2	(AT)7	14	63588	63601
86	p4	(CTAA)3	12	64938	64949
87	p1	(A)11	11	65333	65343
88	p2	(AT)5	10	67294	67303
89	p1	(T)10	10	67350	67359
90	p4	(AGAA)3	12	67660	67671
91	p1	(A)11	11	68918	68928
92	p2	(AT)6	12	69475	69486
93	p4	(CATA)3	12	70764	70775

94	p1	(T)10	10	70930	70939
95	p1	(A)10	10	71564	71573
96	p3	(TTC)4	12	72016	72027
97	p1	(A)10	10	72799	72808
98	p3	(AGA)4	12	72891	72902
99	p1	(T)18	18	72938	72955
100	p1	(T)10	10	72987	72996
101	p2	(TA)7	14	74299	74312
102	p1	(T)12	12	75048	75059
103	p1	(T)10	10	75312	75321
104	p4	(ATAA)3	12	75508	75519
105	p1	(A)10	10	75842	75851
106	p1	(T)12	12	76034	76045
107	p1	(A)13	13	79390	79402
108	p2	(AT)5	10	79967	79976
109	p1	(A)15	15	80107	80121
110	p2	(TA)5	10	81896	81905
111	p1	(T)10	10	84362	84371
112	p3	(GGA)4	12	84931	84942
113	p1	(T)11	11	85433	85443
114	p1	(T)10	10	86672	86681
115	p1	(T)12	12	86816	86827
116	p4	(TTCT)3	12	87161	87172
117	p1	(T)10	10	89232	89241
118	p2	(GA)5	10	94943	94952
119	p6	(TGATAG)3	18	96784	96801
120	p6	(GAAGAG)3	18	97919	97936
121	p2	(TA)5	10	99894	99903
122	p1	(T)11	11	104518	104528
123	p1	(T)10	10	116982	116991
124	p4	(ATTT)3	12	117753	117764
125	p4	(GAAT)3	12	118504	118515
126	p4	(AATT)3	12	119087	119098
127	c*	(TAA)4(A)14*	24	121266	121289
128	p1	(A)11	11	122000	122010
129	p2	(TA)6	12	122137	122148
130	p1	(T)11	11	122330	122340
131	p4	(AATA)3	12	124315	124326
132	p2	(TA)6	12	126474	126485
133	p2	(TA)6	12	126496	126507
134	p2	(AT)6	12	126529	126540
135	p1	(T)10	10	127783	127792
136	p1	(T)19	19	129309	129327
137	p4	(AAAT)3	12	129402	129413
138	p1	(A)10	10	129880	129889
139	p1	(A)10	10	131740	131749
140	p1	(T)15	15	132093	132107
141	p2	(AT)5	10	132152	132161

	142	p1	(T)10	10	132244	132253
	143	p1	(T)11	11	133477	133487
	144	p1	(T)12	12	133610	133621
	145	p1	(T)11	11	133743	133753
	146	p2	(AT)5	10	134078	134087
	147	p4	(CATT)3	12	134352	134363
	148	p4	(AAAT)3	12	135104	135115
	149	p1	(A)10	10	135877	135886
	150	p1	(A)11	11	148340	148350
	151	p2	(AT)5	10	152964	152973
	152	p6	(TCCTCT)3	18	154930	154947
	153	p6	(TCACTA)3	18	156064	156081
	154	p2	(TC)5	10	157916	157925
	155	p1	(A)10	10	163627	163636
A. villosum var. *xanthioides*	1	p3	(GCT)4	12	721	732
	2	p1	(T)10	10	1689	1698
	3	p2	(AT)5	10	2663	2672
	4	p2	(AT)5	10	3444	3453
	5	p1	(A)11	11	3696	3706
	6	p1	(T)11	11	3987	3997
	7	p1	(A)10	10	4341	4350
	8	p1	(A)10	10	4712	4721
	9	p2	(TA)6	12	4933	4944
	10	p1	(T)11	11	4965	4975
	11	p1	(A)17	17	5323	5339
	12	p1	(A)10	10	5681	5690
	13	p1	(T)10	10	5692	5701
	14	p2	(TA)6	12	6196	6207
	15	p1	(A)11	11	6300	6310
	16	p2	(TA)8	16	6650	6665
	17	p1	(A)10	10	7249	7258
	18	p1	(A)11	11	7507	7517
	19	p2	(AT)5	10	7569	7578
	20	p1	(A)11	11	7746	7756
	21	p1	(T)10	10	8681	8690
	22	p5	(TTAAA)3	15	9304	9318
	23	p1	(T)11	11	10017	10027
	24	p1	(A)10	10	10697	10706
	25	p4	(TTAT)3	12	13000	13011
	26	p1	(T)10	10	13179	13188
	27	p1	(T)10	10	13191	13200
	28	p1	(A)10	10	13234	13243
	29	p1	(T)10	10	13915	13924
	30	p4	(TTTA)3	12	13944	13955
	31	p4	(AAAG)3	12	14153	14164
	32	p1	(A)10	10	14914	14923
	33	p6	(AATATA)3	18	15034	15051
	34	p5	(ATATA)3	15	15070	15084
	35	p2	(AT)6	12	15184	15195

36	p1	(T)11	11	16709	16719
37	p1	(T)10	10	17528	17537
38	p1	(T)10	10	19637	19646
39	p1	(T)12	12	19742	19753
40	p1	(A)10	10	19885	19894
41	p2	(AT)5	10	21113	21122
42	p1	(T)11	11	24183	24193
43	p2	(TA)5	10	28644	28653
44	p1	(A)11	11	28851	28861
45	p1	(A)11	11	29058	29068
46	p1	(T)14	14	29463	29476
47	p1	(T)10	10	29564	29573
48	p1	(A)12	12	29926	29937
49	p1	(A)12	12	33148	33159
50	p2	(AT)5	10	34284	34293
51	p3	(TCT)4	12	35052	35063
52	p1	(T)11	11	35094	35104
53	p1	(T)10	10	35333	35342
54	p1	(T)10	10	38268	38277
55	p2	(TA)7	14	38606	38619
56	p1	(T)10	10	39042	39051
57	p1	(A)11	11	39276	39286
58	p1	(A)11	11	39489	39499
59	p1	(A)11	11	47190	47200
60	p1	(A)15	15	47454	47468
61	p5	(AATCA)3	15	48094	48108
62	p1	(T)11	11	49775	49785
63	p2	(TA)7	14	49841	49854
64	p2	(TA)5	10	49872	49881
65	p1	(A)11	11	50231	50241
66	p2	(AT)8	16	50391	50406
67	p4	(CAAA)3	12	50505	50516
68	p1	(T)10	10	52714	52723
69	p1	(T)10	10	53460	53469
70	p3	(ATA)5	15	54349	54363
71	p4	(TAAT)3	12	54399	54410
72	p1	(A)10	10	54566	54575
73	p1	(A)10	10	55341	55350
74	p4	(ATTT)3	12	56323	56334
75	p1	(T)11	11	58803	58813
76	p3	(AAG)4	12	59121	59132
77	p1	(T)10	10	59293	59302
78	p1	(A)12	12	60942	60953
79	p1	(T)11	11	61628	61638
80	p1	(A)10	10	63237	63246
81	p2	(AT)7	14	63282	63295
82	p1	(T)11	11	63368	63378
83	p2	(AT)8	16	63503	63518

84	p4	(CTAA)3	12	64855	64866
85	p1	(A)11	11	65250	65260
86	p2	(AT)5	10	67211	67220
87	p1	(T)11	11	67267	67277
88	p4	(AGAA)3	12	67578	67589
89	p1	(A)11	11	68836	68846
90	p1	(T)10	10	69032	69041
91	p2	(TA)8	16	69395	69410
92	p1	(A)10	10	69696	69705
93	p4	(CATA)3	12	70688	70699
94	p1	(T)11	11	70854	70864
95	p3	(TTC)4	12	71939	71950
96	p1	(A)10	10	72722	72731
97	p3	(AGA)4	12	72814	72825
98	p1	(A)10	10	72846	72855
99	p1	(T)17	17	72862	72878
100	p2	(TA)7	14	74221	74234
101	p1	(T)19	19	74971	74989
102	p1	(T)12	12	75242	75253
103	p4	(ATAA)3	12	75440	75451
104	p1	(T)10	10	75966	75975
105	p1	(A)11	11	79320	79330
106	p2	(AT)5	10	79895	79904
107	p1	(A)13	13	80035	80047
108	p2	(TA)5	10	81822	81831
109	p3	(GGA)4	12	84855	84866
110	p1	(T)12	12	85357	85368
111	p1	(T)10	10	86597	86606
112	p4	(TTCT)3	12	87083	87094
113	p1	(T)11	11	89154	89164
114	p2	(GA)5	10	94862	94871
115	p6	(TGATAG)3	18	96703	96720
116	p6	(GAAGAG)3	18	97838	97855
117	p2	(TA)5	10	99813	99822
118	p1	(T)11	11	104437	104447
119	p1	(A)10	10	111071	111080
120	p1	(T)10	10	116901	116910
121	p4	(ATTT)3	12	117672	117683
122	p4	(GAAT)3	12	118423	118434
123	p4	(AATT)3	12	119007	119018
124	c*	(TAA)5(A)12*	25	121186	121210
125	p1	(A)11	11	121921	121931
126	p2	(TA)6	12	122058	122069
127	p1	(T)13	13	122251	122263
128	p4	(AATA)3	12	124238	124249
129	p2	(TA)6	12	126397	126408
130	p2	(TA)6	12	126419	126430
131	p2	(AT)6	12	126452	126463
132	p1	(T)10	10	127707	127716

	133	p1	(A)10	10	129137	129146
	134	p1	(T)22	22	129232	129253
	135	p4	(AAAT)3	12	129321	129332
	136	p1	(A)10	10	131657	131666
	137	p1	(T)11	11	132015	132025
	138	p2	(AT)5	10	132070	132079
	139	p1	(T)11	11	132162	132172
	140	p1	(T)11	11	133396	133406
	141	p1	(T)12	12	133529	133540
	142	p1	(T)11	11	133662	133672
	143	p2	(AT)5	10	133997	134006
	144	p4	(CATT)3	12	134271	134282
	145	p4	(AAAT)3	12	135023	135034
	146	p1	(A)10	10	135796	135805
	147	p1	(T)10	10	141626	141635
	148	p1	(A)11	11	148259	148269
	149	p2	(AT)5	10	152883	152892
	150	p6	(TCCTCT)3	18	154849	154866
	151	p6	(TCACTA)3	18	155983	156000
	152	p2	(TC)5	10	157835	157844
	153	p1	(A)11	11	163542	163552
A. longiligulare	1	p3	(GCT)4	12	706	717
	2	p2	(AT)5	10	2672	2681
	3	p2	(AT)5	10	3453	3462
	4	p1	(A)10	10	3700	3709
	5	p1	(T)11	11	3991	4001
	6	p5	(AATAA)3	15	4009	4023
	7	p1	(A)11	11	4345	4355
	8	p1	(T)10	10	4434	4443
	9	p1	(A)10	10	4718	4727
	10	p2	(TA)5	10	4916	4925
	11	p2	(TA)7	14	4933	4946
	12	p1	(A)22	22	5307	5328
	13	p1	(A)13	13	5668	5680
	14	p2	(TA)8	16	6191	6206
	15	p1	(A)12	12	6299	6310
	16	p2	(TA)9	18	6651	6668
	17	p1	(A)10	10	7268	7277
	18	p1	(A)12	12	7525	7536
	19	p2	(AT)5	10	7588	7597
	20	p1	(A)10	10	7765	7774
	21	p1	(T)10	10	8699	8708
	22	p5	(TTAAA)3	15	9352	9366
	23	p1	(T)10	10	10071	10080
	24	p1	(A)10	10	10751	10760
	25	p4	(TTAT)3	12	13054	13065
	26	p1	(T)11	11	13233	13243
	27	p1	(T)10	10	13246	13255

	28	p1	(A)10	10	13288	13297
	29	p1	(T)10	10	13969	13978
	30	p1	(T)10	10	13984	13993
	31	p4	(TTTA)3	12	13998	14009
	32	p4	(AAAG)3	12	14206	14217
	33	p1	(A)12	12	14967	14978
	34	p5	(ATATA)3	15	15125	15139
	35	p2	(AT)6	12	15239	15250
	36	p1	(T)11	11	16756	16766
	37	p1	(T)10	10	17575	17584
	38	p1	(T)10	10	19684	19693
	39	p1	(T)12	12	19789	19800
	40	p1	(A)10	10	19932	19941
	41	p2	(AT)5	10	21160	21169
	42	p1	(T)12	12	24212	24223
	43	p2	(TA)5	10	28677	28686
	44	p1	(A)14	14	28883	28896
	45	p1	(A)11	11	29093	29103
	46	p1	(T)13	13	29498	29510
	47	p1	(T)14	14	29598	29611
	48	p1	(A)11	11	29981	29991
	49	p1	(A)11	11	30961	30971
	50	p1	(A)10	10	32644	32653
	51	p1	(A)14	14	33198	33211
	52	p1	(T)10	10	33792	33801
	53	p2	(AT)5	10	34320	34329
	54	p3	(TCT)4	12	35080	35091
	55	p1	(T)11	11	35122	35132
	56	p1	(T)19	19	35361	35379
	57	p1	(T)10	10	38299	38308
	58	p2	(TA)10	20	38642	38661
	59	p1	(T)12	12	39084	39095
	60	p1	(A)10	10	39119	39128
	61	p1	(A)10	10	39319	39328
	62	p1	(A)10	10	39535	39544
	63	p1	(T)11	11	39570	39580
	64	p1	(A)11	11	47241	47251
	65	p1	(A)14	14	47505	47518
	66	p5	(AATCA)3	15	48144	48158
	67	p1	(T)14	14	49813	49826
	68	p2	(TA)9	18	49882	49899
	69	p2	(TA)6	12	50115	50126
	70	p1	(A)14	14	50237	50250
	71	p2	(AT)9	18	50400	50417
	72	p4	(CAAA)3	12	50516	50527
	73	p1	(T)10	10	51831	51840
	74	p1	(T)12	12	52717	52728
	75	p1	(T)10	10	53465	53474
	76	p3	(ATA)5	15	54360	54374

77	p4	(TAAT)3	12	54410	54421
78	p1	(A)10	10	54577	54586
79	p1	(A)10	10	55331	55340
80	p4	(ATTT)3	12	56312	56323
81	p1	(T)12	12	58792	58803
82	p1	(T)14	14	59283	59296
83	p1	(A)10	10	60936	60945
84	p1	(A)16	16	63213	63228
85	p2	(AT)7	14	63264	63277
86	c	(TG)5(T)14	24	63355	63378
87	p2	(AT)7	14	63523	63536
88	p4	(CTAA)3	12	64874	64885
89	p1	(A)11	11	65269	65279
90	p2	(AT)5	10	67230	67239
91	p1	(T)13	13	67286	67298
92	p4	(AGAA)3	12	67599	67610
93	p2	(AT)7	14	69398	69411
94	p1	(A)11	11	69699	69709
95	p4	(CATA)3	12	70692	70703
96	p1	(T)11	11	70858	70868
97	p5	(TTTAT)3	15	71118	71132
98	p3	(TTC)4	12	71949	71960
99	p1	(A)10	10	72732	72741
100	p3	(AGA)4	12	72824	72835
101	p1	(T)21	21	72871	72891
102	p1	(T)11	11	72923	72933
103	p2	(TA)7	14	74236	74249
104	p1	(T)13	13	74985	74997
105	p1	(T)12	12	75250	75261
106	p4	(ATAA)3	12	75448	75459
107	p1	(T)12	12	75716	75727
108	p1	(T)10	10	75976	75985
109	p1	(A)12	12	79323	79334
110	p2	(AT)5	10	79899	79908
111	p2	(TA)5	10	81786	81795
112	p3	(GGA)4	12	84817	84828
113	p1	(T)10	10	85319	85328
114	p1	(T)10	10	86557	86566
115	p4	(TTCT)3	12	87042	87053
116	p1	(T)10	10	89114	89123
117	p2	(GA)5	10	94688	94697
118	p6	(TGATAG)3	18	96529	96546
119	p2	(TA)5	10	99625	99634
120	p1	(T)11	11	104241	104251
121	p1	(T)10	10	116705	116714
122	p4	(GAAT)3	12	118227	118238
123	p4	(AATT)3	12	118859	118870
124	p1	(A)14	14	121038	121051

	125	p1	(A)10	10	121754	121763
	126	p2	(TA)7	14	121898	121911
	127	p1	(T)12	12	122094	122105
	128	p4	(AATA)3	12	124080	124091
	129	p2	(TA)6	12	126229	126240
	130	p2	(TA)6	12	126251	126262
	131	p2	(AT)6	12	126284	126295
	132	p1	(T)10	10	127539	127548
	133	p1	(A)11	11	128811	128821
	134	p1	(A)12	12	128972	128983
	135	p1	(T)18	18	129068	129085
	136	p4	(AAAT)3	12	129153	129164
	137	p1	(T)11	11	129305	129315
	138	p1	(A)10	10	129633	129642
	139	p1	(A)10	10	131493	131502
	140	p1	(T)14	14	131842	131855
	141	p2	(AT)5	10	131894	131903
	142	p1	(T)12	12	133308	133319
	143	p1	(T)11	11	133441	133451
	144	p2	(AT)5	10	133776	133785
	145	p4	(CATT)3	12	134050	134061
	146	p1	(A)10	10	135575	135584
	147	p1	(A)11	11	148038	148048
	148	p2	(AT)5	10	152654	152663
	149	p6	(TCACTA)3	18	155740	155757
	150	p2	(TC)5	10	157592	157601
	151	p1	(A)10	10	163166	163175

Table S4. Simple sequence repeats (SSRs) in protein-coding regions of the chloroplast genome of *A. villosum, A. villosum* var. *xanthioides* and *A. longiligulare.*

Species	Regions	SSR nr.	SSR type	SSR	size	start	end
A. villosum	*cemA*	1	p1	(A)11	11	4	14
	clpP	1	p4	(TTTA)3	12	185	196
	matK	1	p2	(TA)5	10	659	668
	ndhD	1	p4	(TATT)3	12	1293	1304
	ndhF	1	p4	(AATT)3	12	1883	1894
	ndhI	1	p1	(A)10	10	487	496
	ndhK	1	p1	(A)10	10	29	38
	psbA	1	p3	(GCA)4	12	454	465
	rpoC2	1	p2	(AT)5	10	752	761
	rpoC2	2	p1	(T)10	10	1980	1989
	rpoC2	3	p1	(A)12	12	2121	2132
	rpoC2	4	p1	(A)10	10	2228	2237
	rps18	1	p1	(A)10	10	324	333
	ycf1	1	p1	(T)10	10	2101	2110
	ycf1	2	p4	(ATTT)3	12	2872	2883
	ycf1	3	p4	(GAAT)3	12	3623	3634
	ycf1	1	p1	(T)10	10	2101	2110
	ycf1	2	p4	(ATTT)3	12	2872	2883
	ycf1	3	p4	(GAAT)3	12	3623	3634
	ycf1	4	p2	(AT)5	10	3900	3909
	ycf1	5	p1	(A)11	11	4234	4244
	ycf1	6	p1	(A)12	12	4366	4377
	ycf1	7	p1	(A)11	11	4500	4510
	ycf2	1	p2	(GA)5	10	3157	3166
	ycf2	2	p6	(TGATAG)3	18	4998	5015
	ycf2	3	p6	(GAAGAG)3	18	6133	6150
	ycf2	1	p2	(GA)5	10	3157	3166
	ycf2	2	p6	(TGATAG)3	18	4998	5015
	ycf2	3	p6	(GAAGAG)3	18	6133	6150
A. villosum var. xanthioides	*cemA*	1	p1	(A)11	11	4	14
	clpP	1	p4	(TTTA)3	12	185	196
	matK	1	p2	(TA)5	10	659	668
	ndhD	1	p4	(TATT)3	12	1293	1304
	ndhF	1	p4	(AATT)3	12	1883	1894
	ndhI	1	p1	(A)10	10	487	496
	ndhK	1	p1	(A)10	10	29	38
	psbA	1	p3	(GCA)4	12	454	465
	rpoC2	1	p2	(AT)5	10	752	761
	rpoC2	2	p1	(T)10	10	1980	1989
	rpoC2	3	p1	(A)12	12	2121	2132
	rpoC2	4	p1	(A)10	10	2228	2237
	rps18	1	p1	(A)10	10	324	333
	ycf1	1	p1	(T)10	10	2101	2110
	ycf1	2	p4	(ATTT)3	12	2872	2883

	ycf1	3	p4	(GAAT)3	12	3623	3634
	ycf1	1	p1	(T)10	10	2101	2110
	ycf1	2	p4	(ATTT)3	12	2872	2883
	ycf1	3	p4	(GAAT)3	12	3623	3634
	ycf1	4	p2	(AT)5	10	3900	3909
	ycf1	5	p1	(A)11	11	4234	4244
	ycf1	6	p1	(A)12	12	4366	4377
	ycf1	7	p1	(A)11	11	4500	4510
	ycf2	1	p2	(GA)5	10	3157	3166
	ycf2	2	p6	(TGATAG)3	18	4998	5015
	ycf2	3	p6	(GAAGAG)3	18	6133	6150
	ycf2	1	p2	(GA)5	10	3157	3166
	ycf2	2	p6	(TGATAG)3	18	4998	5015
	ycf2	3	p6	(GAAGAG)3	18	6133	6150
A. longiligulare	*cemA*	1	p1	(A)11	11	4	14
	clpP	1	p4	(TTTA)3	12	185	196
	matK	1	p2	(TA)5	10	659	668
	ndhD	1	p4	(TATT)3	12	1293	1304
	ndhF	1	p4	(AATT)3	12	1883	1894
	ndhI	1	p1	(A)10	10	487	496
	ndhK	1	p1	(A)10	10	29	38
	psbA	1	p3	(GCA)4	12	454	465
	rpoC2	1	p2	(AT)5	10	752	761
	rpoC2	2	p1	(T)10	10	1980	1989
	rpoC2	3	p1	(A)12	12	2121	2132
	rpoC2	4	p1	(A)10	10	2228	2237
	rps18	1	p1	(A)10	10	324	333
	ycf1	1	p1	(T)10	10	2101	2110
	ycf1	2	p4	(ATTT)3	12	2872	2883
	ycf1	3	p4	(GAAT)3	12	3623	3634
	ycf1	1	p1	(T)10	10	2101	2110
	ycf1	2	p4	(ATTT)3	12	2872	2883
	ycf1	3	p4	(GAAT)3	12	3623	3634
	ycf1	4	p2	(AT)5	10	3900	3909
	ycf1	5	p1	(A)11	11	4234	4244
	ycf1	6	p1	(A)12	12	4366	4377
	ycf1	7	p1	(A)11	11	4500	4510
	ycf2	1	p2	(GA)5	10	3157	3166
	ycf2	2	p6	(TGATAG)3	18	4998	5015
	ycf2	3	p6	(GAAGAG)3	18	6133	6150
	ycf2	1	p2	(GA)5	10	3157	3166
	ycf2	2	p6	(TGATAG)3	18	4998	5015
	ycf2	3	p6	(GAAGAG)3	18	6133	6150

Table S5. Sample information of species in *Amomum*.

No.	Species	Sample number	Sample	Use	Location
1	*A. villosum*	Y17085MT01	Fresh leaf	Cp genome sequencing	Haikou, Hainan Province, China
2	*A. villosum* var. *xanthioides*	Y17088MT01	Fresh leaf	Cp genome sequencing	Xishuangbanna, Yunnan Province, China
3	*A. villosum*	Y17089MT01	Fresh leaf	Cp genome sequencing	Yangchun, Guangdong Province, China
4	*A. villosum*	Y19017MT01	Fresh leaf	Cp genome sequencing	Xishuangbanna, Yunnan Province, China
5	*A. villosum* var. *xanthioides*	Y19018MT01	Fresh leaf	Cp genome sequencing	Xishuangbanna, Yunnan Province, China
6	*A. longiligulare*	Y19019MT01	Fresh leaf	Cp genome sequencing	Guangzhou, Guangdong Province, China
7	*A. longiligulare*	Y19020MT01	Fresh leaf	Cp genome sequencing	Haikou, Hainan Province, China
8	*A. villosum*	Y19021MT01	Fresh leaf	Cp genome sequencing	Nanning, Guangxi Province, China
9	*A. villosum*	Y19005MT01	Dry leaf	Primer validation	Yangchun, Guangdong Province, China
10	*A. villosum*	Y19005MT02	Dry leaf	Primer validation	Yangchun, Guangdong Province, China
11	*A. villosum*	Y19005MT03	Dry leaf	Primer validation	Yangchun, Guangdong Province, China
12	*A. villosum*	Y19005MT04	Dry leaf	Primer validation	Yangchun, Guangdong Province, China
13	*A. villosum*	Y19005MT05	Dry leaf	Primer validation	Yangchun, Guangdong Province, China
14	*A. longiligulare*	Y19006MT01	Dry leaf	Primer validation	Guangzhou, Guangdong Province, China
15	*A. longiligulare*	Y19006MT02	Dry leaf	Primer validation	Guangzhou, Guangdong Province, China
16	*A. longiligulare*	Y19006MT03	Dry leaf	Primer validation	Guangzhou, Guangdong Province, China
17	*A. longiligulare*	Y19006MT04	Dry leaf	Primer validation	Guangzhou, Guangdong Province, China
18	*A. longiligulare*	Y19006MT05	Dry leaf	Primer validation	Guangzhou, Guangdong Province, China
19	*A. longiligulare*	Y19007MT01	Dry leaf	Primer validation	Haikou, Hainan Province, China
20	*A. longiligulare*	Y19007MT02	Dry leaf	Primer validation	Haikou, Hainan Province, China
21	*A. chinense*	Y19008MT01	Dry leaf	Primer validation	Haikou, Hainan Province, China
22	*A. chinense*	Y19008MT02	Dry leaf	Primer validation	Haikou, Hainan Province, China
23	*A. chinense*	Y19008MT03	Dry leaf	Primer validation	Haikou, Hainan Province, China
24	*A. ompactum*	Y19009MT01	Dry leaf	Primer validation	Haikou, Hainan Province, China
25	*A. ompactum*	Y19009MT02	Dry leaf	Primer validation	Haikou, Hainan Province, China

26	*A. ompactum*	Y19009MT03	Dry leaf	Primer validation	Haikou, Hainan Province, China
27	*A. saoko*	Y19010MT01	Dry leaf	Primer validation	Nanning, Guangxi Province, China
28	*A. saoko*	Y19010MT02	Dry leaf	Primer validation	Nanning, Guangxi Province, China
29	*A. saoko*	Y19010MT03	Dry leaf	Primer validation	Nanning, Guangxi Province, China
30	*A. villosum*	Y19011MT01	Dry leaf	Primer validation	Xishuangbanna, Yunnan Province, China
31	*A. villosum*	Y19011MT02	Dry leaf	Primer validation	Xishuangbanna, Yunnan Province, China
32	*A. villosum*	Y19011MT03	Dry leaf	Primer validation	Xishuangbanna, Yunnan Province, China
33	*A. villosum* var. *xanthioides*	Y19012MT01	Dry leaf	Primer validation	Xishuangbanna, Yunnan Province, China
34	*A. villosum* var. *xanthioides*	Y19012MT02	Dry leaf	Primer validation	Xishuangbanna, Yunnan Province, China
35	*A. koenigii*	Y19013MT01	Dry leaf	Primer validation	Xishuangbanna, Yunnan Province, China
36	*A. koenigii*	Y19013MT02	Dry leaf	Primer validation	Xishuangbanna, Yunnan Province, China
37	*A. koenigii*	Y19013MT03	Dry leaf	Primer validation	Xishuangbanna, Yunnan Province, China
38	*A. maximum*	Y19014MT01	Dry leaf	Primer validation	Xishuangbanna, Yunnan Province, China
39	*A. maximum*	Y19014MT02	Dry leaf	Primer validation	Xishuangbanna, Yunnan Province, China
40	*A. maximum*	Y19014MT03	Dry leaf	Primer validation	Xishuangbanna, Yunnan Province, China

Table S6. Primer sequences of the highly divergent regions, including *trnD-trnY*, *accD-psaI*, *ycf4-cemA*, and *trnI-ycf*2.

Regions	Forward/Reverse	Primer sequence (5' to 3')
atpH-atpI-1	F	TGGTAAGTTCCTCGCACAAAAC
	R	TAAGCTCTAAACCCCCGTCT
atpH-atpI-2	F	AGATCCAGCGGTCAAAGTAAC
	R	AGGGCTGGGGAGGTTATAGTT
trnD-trnY,	F	TCGTCGTGTTGTAAGAGAGACAT
	R	TCCTTCTCTTTTACGGATCAAGCA
accD-psaI	F	GACAGTACCCGAGGGTTCAC
	R	TTGCCGGAAATACTAGGCCC
ycf4-cemA	F	ATCCTCTCGAGGTGGGGAAA
	R	TTCTCTGAAGCACGAGTTGT
trnI-ycf2	F	GGGCGCTTTAACCATTCAGC
	R	TATTTAGACCTCCTCCGGCGA

Table S7. Primer sequences at the boundaries between single copy and IR regions.

Species	Regions	Forward/Reverse	Primer sequence (5' to 3')
A. villosum	LSC-IRa	F	CACGCTCGATAAGGCATGAG
		R	ACTTGGTCTCGGGCATCTAC
	IRa-SSC	F	TCGCGGTACCCATCCTTTTA
		R	TGGGGTTGTTGTTGCAAGTT
	SSC-IRb	F	TCGATTCATTCCCATCCAATCA
		R	CTTCCTTGTCCCAAGCATATGT
	IRb-LSC	F	ACTTGGTCTCGGGCATCTAC
		R	CCGTAGTTGACAGTCAGGGT
A. villosum var. *xanthioides*	LSC-IRa	F	CACGCTCGATAAGGCATGAG
		R	ACTTGGTCTCGGGCATCTAC
	IRa-SSC	F	TCGCGGTACCCATCCTTTTA
		R	TGGGGTTGTTGTTGCAAGTT
	SSC-IRb	F	TCGATTCATTCCCATCCAATCA
		R	ACCCGAGGAATATTGAACTCCA
	IRb-LSC	F	ACTTGGTCTCGGGCATCTAC
		R	CCGTAGTTGACAGTCAGGGT
A. longiligulare	LSC-IRa	F	CACGCTCGATAAGGCATGAG
		R	ACTTGGTCTCGGGCATCTAC
	IRa-SSC	F	TCGCGGTACCCATCCTTTTA
		R	TGGGGTTGTTGTTGCAAGTT
	SSC-IRb	F	TCGATTCATTCCCATCCAATCA
		R	CTTCCTTGTCCCAAGCATATGT
	IRb-LSC	F	ACTTGGTCTCGGGCATCTAC
		R	CCGTAGTTGACAGTCAGGGT

Table S8. GenBank accession numbers of complete chloroplast genome sequences used for phylogenetic analyses.

GenBank acc.	Species	GenBank acc.	Species
NC_001879	*Nicotiana tabacum*	NC_027477	*Melica mutica*
JX_088669	*Xiphidium caeruleum*	NC_028439	*Musa balbisiana*
NC_002762	*Triticum aestivum*	NC_028729	*Curcuma flaviflora*
NC_008590	*Hordeum vulgare subsp. vulgare*	NC_029948	*Caryota mitis*
NC_020362	*Heliconia collinsiana*	NC_029954	*Salacca ramosiana*
NC_020363	*Zingiber spectabile*	NC_029958	*Nypa fruticans*
NC_020364	*Pseudophoenix vinifera*	NC_029962	*Hanguana malayana*
NC_020365	*Calamus caryotoides*	NC_029965	*Corypha lecomtei*
NC_020367	*Dasypogon bromeliifolius*	NC_029970	*Baxteria australis*
NC_020431	*Salvia miltiorrhiza*	NC_029971	*Arenga caudata*
NC_021761	*Secale cereale*	NC_029972	*Areca vestiaria*
NC_022417	*Cocos nucifera*	NC_031333	*Oryza sativa*
NC_022926	*Musa textilis*	NC_031427	*Joinvillea ascendens*
NC_022928	*Curcuma roscoeana*	NC_035895	*Alpinia oxyphylla*
NC_024830	*Aegilops longissima*	NC_036935	*Amomum krervanh*
NC_027468	*Avena sativa*	NC_036992	*Amomum compactum*
NC_027472	*Bromus vulgaris*	NC_037026	*Stipa capillata*
NC_026220	*Ananas comosus*		
